# Anaphylactic and nonanaphylactic reactions to SARS-CoV-2 vaccines: a systematic review and meta-analysis

**DOI:** 10.1186/s13223-021-00613-7

**Published:** 2021-10-16

**Authors:** Saad Alhumaid, Abbas Al Mutair, Zainab Al Alawi, Ali A. Rabaan, Raghavendra Tirupathi, Mohammed A. Alomari, Aqeel S. Alshakhes, Abeer M. Alshawi, Gasmelseed Y. Ahmed, Hassan M. Almusabeh, Tariq T. Alghareeb, Abdulaziz A. Alghuwainem, Zainab A. Alsulaiman, Mohammed A. Alabdulmuhsin, Emad A. AlBuwaidi, Amjad K. Bu Dukhi, Hani N. Mufti, Manaf Al-Qahtani, Kuldeep Dhama, Jaffar A. Al-Tawfiq, Awad Al-Omari

**Affiliations:** 1 Administration of Pharmaceutical Care, Al-Ahsa Health Cluster, Ministry of Health, Rashdiah Street, P. O. Box 12944, Al-Ahsa, 31982 Saudi Arabia; 2 Research Center, Almoosa Specialist Hospital, Al-Ahsa, Saudi Arabia; 3 College of Nursing, Princess Norah Bint Abdul Rahman University, Riyadh, Saudi Arabia; 4 School of Nursing, University of Wollongong, Wollongong, Australia; 5 Division of Allergy and Immunology, College of Medicine, King Faisal University, Al-Ahsa, Saudi Arabia; 6 Molecular Diagnostic Laboratory, Johns Hopkins Aramco Healthcare, Dhahran, Saudi Arabia; 7 Department of Public Health and Nutrition, The University of Haripur, Haripur, 22610 Pakistan; 8 Department of Medicine Keystone Health, Penn State University School of Medicine, Hershey, PA USA; 9 Department of Medicine, Wellspan Chambersburg and Waynesboro (Pa.) Hospitals, Chambersburg, PA USA; 10 Palliative Care Department, King Fahad Medical City, Riyadh, Saudi Arabia; 11 Department of Psychiatry, Prince Saud Bin Jalawi Hospital, Al-Ahsa, Saudi Arabia; 12 Department of Pharmacy, King Fahad Hofuf Hospital, Al-Ahsa, Saudi Arabia; 13 Administration of Compliance, Al-Ahsa Health Affairs, Ministry of Health, Al-Ahsa, Saudi Arabia; 14 Department of Pharmacy, Hereditary Blood Diseases Centre, Al-Ahsa, Saudi Arabia; 15 Department of Pharmacy, Maternity and Children Hospital, Al-Ahsa, Saudi Arabia; 16 Primary Care Medicine, Al-Ahsa Health Cluster, Ministry of Health, Al-Ahsa, Saudi Arabia; 17 Department of Pharmacy, Prince Saud Bin Jalawi Hospital, Al-Ahsa, Saudi Arabia; 18 Department of Cardiac Sciences, King Abdulaziz Medical City, Ministry of National Guard Health Affairs, Jeddah, Saudi Arabia; 19 Department Cardiac Sciences, College of Medicine, King Saud bin Abdulaziz University for Health Sciences, Ministry of National Guard Health Affairs, Jeddah, Saudi Arabia; 20 Department of Medical Research, King Abdullah International Medical Research Center, Ministry of National Guard Health Affairs, Jeddah, Saudi Arabia; 21 Department of Medicine, Royal College of Surgeons in Ireland, Bahrain, Kingdom of Bahrain; 22 Department of Infectious Diseases, Royal Medical Services, Bahrain Defence Force Hospital, Riffa, Kingdom of Bahrain; 23 Division of Pathology, ICAR-Indian Veterinary Research Institute, Izatnagar, Bareilly, Uttar Pradesh 243122 India; 24 Infectious Disease Unit, Specialty Internal Medicine, Johns Hopkins Aramco Healthcare, Dhahran, Saudi Arabia; 25 Infectious Disease Division, Department of Medicine, Indiana University School of Medicine, Indianapolis, IN USA; 26 Infectious Disease Division, Department of Medicine, Johns Hopkins University School of Medicine, Baltimore, MD USA; 27 College of Medicine, Alfaisal University, Riyadh, Saudi Arabia; 28 Research Center, Dr. Sulaiman Al Habib Medical Group, Riyadh, Saudi Arabia

**Keywords:** Allergic, COVID-19, Immunologic, Incidence, Reactions, SARS-Cov-2, Systematic review, Vaccines

## Abstract

**Background:**

Currently there is no systematic review and meta-analysis of the global incidence rates of anaphylactic and nonanaphylactic reactions to SARS-CoV-2 vaccines in the general adult population.

**Objectives:**

To estimate the incidence rates of anaphylactic and nonanaphylactic reactions after COVID-19 vaccines and describe the demographic and clinical characteristics, triggers, presenting signs and symptoms, treatment and clinical course of confirmed cases.

**Design:**

A systematic review and meta-analysis. Preferred Reporting Items for Systematic Reviews and Meta-Analyses [PRISMA] statement was followed.

**Methods:**

Electronic databases (Proquest, Medline, Embase, Pubmed, CINAHL, Wiley online library, and Nature) were searched from 1 December 2020 to 31 May 2021 in the English language using the following keywords alone or in combination: *anaphylaxis*, *non-anaphylaxis*, *anaphylactic reaction*, *nonanaphylactic reaction*, *anaphylactic/anaphylactoid shock*, *hypersensitivity*, *allergy reaction*, *allergic reaction*, *immunology reaction*, *immunologic reaction*, *angioedema*, *loss of consciousness*, *generalized erythema*, *urticaria*, *urticarial rash*, *cyanosis*, *grunting*, *stridor*, *tachypnoea*, *wheezing*, *tachycardia*, *abdominal pain*, *diarrhea*, *nausea*, *vomiting* and *tryptase*. We included studies in adults of all ages in all healthcare settings. Effect sizes of prevalence were pooled with 95% confidence intervals (CIs). To minimize heterogeneity, we performed sub-group analyses.

**Results:**

Of the 1,734 papers that were identified, 26 articles were included in the systematic review (8 case report, 5 cohort, 4 case series, 2 randomized controlled trial and 1 randomized cross-sectional studies) and 14 articles (1 cohort, 2 case series, 1 randomized controlled trial and 1 randomized cross-sectional studies) were included in meta-analysis. Studies involving 26,337,421 vaccine recipients [Pfizer-BioNTech (n = 14,505,399) and Moderna (n = 11,831,488)] were analyzed. The overall pooled prevalence estimate of anaphylaxis to both vaccines was 5.0 (95% CI 2.9 to 7.2, *I*^*2*^ = 81%, *p* =  < 0.0001), while the overall pooled prevalence estimate of nonanaphylactic reactions to both vaccines was 53.9 (95% CI 0.0 to 116.1, *I*^*2*^ = 99%, *p* =  < 0.0001). Vaccination with Pfizer-BioNTech resulted in higher anaphylactic reactions compared to Moderna (8.0, 95% CI 0.0 to 11.3, *I*^*2*^ = 85% versus 2.8, 95% CI 0.0 to 5.7, *I*^*2*^ = 59%). However, lower incidence of nonanaphylactic reactions was associated with Pfizer-BioNTech compared to Moderna (43.9, 95% CI 0.0 to 131.9, *I*^*2*^ = 99% versus 63.8, 95% CI 0.0 to 151.8, *I*^*2*^ = 98%). The funnel plots for possible publication bias for the pooled effect sizes to determine the incidence of anaphylaxis and nonanaphylactic reactions associated with mRNA COVID-19 immunization based on mRNA vaccine type appeared asymmetrical on visual inspection, and Egger’s tests confirmed asymmetry by producing *p* values < 0.05. Across the included studies, the most commonly identified risk factors for anaphylactic and nonanaphylactic reactions to SARS-CoV-2 vaccines were female sex and personal history of atopy. The key triggers to anaphylactic and nonanaphylactic reactions identified in these studies included foods, medications, stinging insects or jellyfish, contrast media, cosmetics and detergents, household products, and latex. Previous history of anaphylaxis; and comorbidities such as asthma, allergic rhinitis, atopic and contact eczema/dermatitis and psoriasis and cholinergic urticaria were also found to be important.

**Conclusion:**

The prevalence of COVID-19 mRNA vaccine-associated anaphylaxis is very low; and nonanaphylactic reactions occur at higher rate, however, cutaneous reactions are largely self-limited. Both anaphylactic and nonanaphylactic reactions should not discourage vaccination.

## Background

Globally, as of 28 May 2021, there have been 168,040,871 confirmed cases of coronavirus disease 2019 (COVID-19), including 3,494,758 deaths, reported to the World Health Organization [1]. Immunization is an important strategy to halt COVID-19 pandemic. Two COVID-19 vaccines were granted emergency use authorization [EUA] by the United States Food and Drug Administration: the Pfizer-BioNTech COVID-19 vaccine and the Moderna COVID-19 vaccine [2, 3]. Other vaccines had also been used or authorized in multiple parts of the world. Vaccination hesitancy is an important obstacle to achieve herd immunity [4–6]. One of the main reasons for vaccine hesitancy is the concerns about vaccine safety, adverse effects, or toxicity.

Adverse reactions to vaccines are commonly reported, but most are not immunologically mediated. Non-immunologically mediated reactions are typically related to the pharmacology of the vaccine and consist of toxic reactions, side effects, and medication interactions [7]. Immunologically mediated reactions include IgE-mediated and T-cell mediated reactions as well as other immunologic mechanisms that occur as a result of allergen exposure. These responses, particularly severe anaphylaxis, rarely occur [8, 9]. Symptoms can begin within minutes of exposure to the allergen [generally within 30 min and less commonly within several hours]; and range from urticaria, swelling, and gastrointestinal upset, to respiratory distress and cardiovascular collapse associated with severe anaphylaxis [9]. Delayed reactions typically occur hours to days after exposure to the antigen trigger, and most typically manifest as a cutaneous reaction [10].

Maintaining public confidence to minimize vaccine hesitancy is crucial as disease outbreaks among the unvaccinated population are likely to occur when herd immunity is compromised. We aimed to estimate the incidence rates of anaphylactic and nonanaphylactic reactions after mRNA COVID-19 vaccines (Pfizer/BioNTech and Moderna) and describe the demographic and clinical characteristics, triggers, presenting signs and symptoms, treatment and clinical course of confirmed cases. We primarily focused this assessment on the Pfizer/BioNTech and Moderna mRNA vaccines given that reports of anaphylactic and nonanaphylactic reactions from adenovirus vaccines beyond clinical trials were limited.

## Methods

### Design

We followed the Preferred Reporting Items for Systematic Reviews and Meta-Analyses guidelines [PRISMA] in conducting this systematic review and meta-analysis [11]. The following electronic databases were searched: PROQUEST, MEDLINE, EMBASE, PUBMED, CINAHL, WILEY ONLINE LIBRARY, and NATURE with Full Text. We used the following keywords: *anaphylaxis* OR *non-anaphylaxis* OR *anaphylactic reaction* OR *nonanaphylactic reaction* OR *anaphylactic*/*anaphylactoid shock* OR *hypersensitivity* OR *allergy reaction* OR *allergic reaction* OR *immunology reaction* OR *immunologic reaction* AND *angioedema* OR *loss of consciousness* OR *generalized erythema* OR *urticaria* OR *urticarial rash* OR *cyanosis* OR *grunting* OR *stridor* OR *tachypnoea* OR *wheezing* OR *tachycardia* OR *abdominal pain* OR *diarrhea* OR *nausea* OR *vomiting* OR *tryptase*. The search was limited to papers published in English between 1 December 2020 and 31 May 2021. Based on the title and abstract of each selected article, we selected those discussing and reporting rates of anaphylactic and nonanaphylactic reactions to mRNA SARS-CoV-2 vaccines. We also utilized backward snowballing to increase the yield of potentially relevant articles.

### Inclusion and exclusion criteria

We retained randomized controlled trials, cohort studies, case reports and case series and excluded other studies. We excluded studies that did not report data on anaphylactic and nonanaphylactic reactions to SARS-CoV-2 mRNA vaccines; or studies that never stated details on identified cases with anaphylactic and nonanaphylactic reactions. We evaluated studies that included all adults as our population of interest who received 1 or more doses of mRNA COVID-19 vaccines and reported evidence on anaphylactic and nonanaphylactic reactions during the period from December 1, 2020 through May 31, 2021.

### Data extraction

Six authors (S.A., A.A., A.R., R.T., M.A.A., and Z.A.) critically reviewed all of the studies retrieved and selected those judged to be the most relevant. The abstracts of all citations were examined thoroughly. Data were extracted from the relevant research studies using key headings, which are noted in Tables 1 and 2, simplifying analysis, and review of the literature. Articles were categorized as randomized controlled trial, case report, case series or cohort studies. Studies were also categorized as a national or international online survey as we sought to describe reports of anaphylactic and nonanaphylactic reactions after mRNA COVID-19 vaccination made to the United States’ Vaccine Adverse Event Reporting System (US-VAERS) and Canadian Ontario’s vaccine safety surveillance system; or categorized as self-reported by vaccine recipients, mostly by healthcare workers, via an online questionnaire conducted to compare the safety of available mRNA COVID-19 vaccines.

**Table 1:** Summary of the characteristics of the included studies with evidence on anaphylactic reactions and SARS-CoV-2 vaccines (n = 12), 2020–2021

Author, year, study location	Study design, setting	Age (years)	Male, n (%)	Past history of allergies or allergic reactions	Past history of anaphylaxis	Vaccine brand and dose	Reaction onset after vaccination [min]	Signs and symptoms	Anaphylaxis level of severity	Tryptase level was measured; AND if elevated^§^	Treatment setting: therapy given for reaction	Epinephrine received, n (%)	Outcome	Skin test performed? [Time from reaction to the skin test]; and result	Assessment of study risk of bias (tool used; finding)	Key findings
Blumenthal et al. 2021 [22], United States	Prospective cohort, multicenter Self-reporting by HCWs after their first dose of an mRNA COVID-19 vaccine through a multipronged approach	Mean (SD), 41 (14)	1 (14.3)	Three (43%) persons had prior allergies or allergic reactions, including to drugs or medical products, foods, and shellfish	One (14.3%) person had a prior episode of anaphylaxis from prior exposures; including to contrast media, sulfa antibiotics, morphine, ciprofloxacin, penicillin, cat dander, peanuts, tree nuts, and shellfish	Pfizer-BioNTech, dose 1	Mean [range], 14 [10–30]	Pruritus, urticaria, and/or angioedema, sensation of throat closure, cough, wheeze, and/or dyspnea, hypotension and/or tachycardia, nausea, vomiting, and/or diarrhea	All cases were classified as Brighton Level 2 (7 [100%]) a Cases met NIAID/FAAN criteria Grade I (3 [43%]) or Grade II (4 [57%]) c	Not measured	One patient was hospitalized in ICU, and four were treated in an ED	6 (86)	All patients had recovered or been discharged home. No deaths from anaphylaxis were reported	–	(NOS; 8)	Allergists and immunologists reviewed the EHRs of HCWs to identify anaphylaxis Incidence of vaccine-triggered anaphylaxis per 106 doses = number of anaphylaxis cases [7]/doses administered [25,929] = 270
Blumenthal et al. 2021 [22], United States	Prospective cohort, multicenter Self-reporting by HCWs after their first dose of an mRNA COVID-19 vaccine through a multipronged approach	Mean (SD), 41 (13)	0 (0)	Seven (78%) persons had prior allergies or allergic reactions, including to drugs or medical products, foods, and shellfish	Four (44%) persons had a prior episode of anaphylaxis from prior exposures; including to contrast media, sulfa antibiotics, morphine, ciprofloxacin, penicillin, cat dander, peanuts, tree nuts, and shellfish	Moderna, dose 1	Mean [range], 19 [1–120]	Pruritus, urticaria, and/or angioedema, sensation of throat closure, cough, wheeze, and/or dyspnea, hypotension and/or tachycardia, nausea, vomiting, and/or diarrhea	All cases were classified as Brighton Level 1 (1 [11%]) or Level 2 (6 [67%]) or Level 3 (2 [22%])^a^ Cases met NIAID/FAAN criteria Grade I (4 [44%]) or Grade II (5 [56%])^c^	Yes; AND was elevated in 1 patient (11)	Five patients were treated in an ED	3 (33)	All patients had recovered or been discharged home. No deaths from anaphylaxis were reported	–	(NOS; 8)	Allergists and immunologists reviewed the EHRs of HCWs to identify anaphylaxis Incidence of vaccine-triggered anaphylaxis per 106 doses = number of anaphylaxis cases [9]/doses administered [38,971] = 231
Frank et al. 2021 [25], United States	Case report	55-year-old female HCW	0 (0)	A history of significant allergies, including to fresh fish, iodine solution, and the rabies vaccine	A history of anaphylactic reactions	Pfizer-BioNTech, dose 1	10 min	Despite premedication with I.V. diphenhydramine and dexamethasone 30 min prior to vaccine, patient experienced hives on chest, sensation of throat closure, dyspnea, and wheezing	Case was classified as Brighton Level 1 ^a^	Not measured	Patient was treated at the ED with I.M. epinephrine and I.V. fluids	1 (100)	Patient was discharged from the hospital with a prescription for a steroid taper and EpiPen, instructions to not receive the second dose of the COVID-19 vaccine	–	(NOS; 6)	Patient’s allergic reaction demonstrated by edema of tongue was refractory requiring an epinephrine infusion for nearly 3 days Case was thought to be secondary to the PEG component of the vaccine
Kadali et al. 2021 [27], United States	A randomized, cross‐sectional study using an independent online survey questionnaire was conducted to collect responses from HCWs	Average age of all participants who completed the survey (n = 432) was 43.76 years	About 10.65% of participants were males	Not reported	Not reported	Moderna, unknown dose	Not reported	Not reported	–	Not measured	Not reported	Not reported	Patient recovered with complete resolution of the suspected adverse drug reaction. No death from anaphylaxis was reported	–	RoB 2; low risk of bias	Incidence of vaccine-triggered anaphylaxis per 10^6^ doses = number of anaphylaxis cases [5]/doses administered [432] = 231
Mathioudakis et al. 2021 [29], United Kingdom	Self-reporting by participants via an online survey conducted to compare the safety of available COVID-19 vaccines	Age groups [40–49; n = 3]; [50–59, n = 2] AND [60–69; n = 2]	4 (57)	Not reported	Not reported	Pfizer-BioNTech, dose 1 (n = 1,673), AND dose 2 (n = 509)	Not reported	Not reported	All episodes of anaphylaxis reported were described as mild or moderate	Not measured	None required hospital admission (only treatment or advice from a HCW outside the hospital), suggesting that they most likely represented anaphylactoid reactions	Not reported	Patients recovered with complete resolution of the suspected adverse drug reaction. No deaths from anaphylaxis were reported	–	(NOS; 6)	Incidence of vaccine-triggered anaphylaxis per 10^6^ doses = number of anaphylaxis cases [7]/doses administered [2182] = 3208 Pfizer-BioNTech vaccine recipients reported a considerably lower incidence of anaphylaxis: [RR (95% CI): 0.19 (0.04–0.94); multivariate logistic regression coefficient = -1.890, p = 0.024]
Mathioudakis et al. 2021 [29], United Kingdom	Self-reporting by participants via an online survey conducted to compare the safety of available COVID-19 vaccines	Age groups [30–39; n = 1] AND [40–49; n = 1]	0 (0)	Not reported	Not reported	Moderna, dose 1 (n = 282), AND dose 2 (n = 1)	Not reported	Not reported	All episodes of anaphylaxis reported were described as mild or moderate	Not measured	None required hospital admission (only treatment or advice from a HCW outside the hospital), suggesting that they most likely represented anaphylactoid reactions	Not reported	Patients recovered with complete resolution of the suspected adverse drug reaction. No deaths from anaphylaxis were reported	–	(NOS; 6)	Incidence of vaccine-triggered anaphylaxis per 10^6^ doses = number of anaphylaxis cases (5)/doses administered [283] = 7067
Ontario Public Health Agency 2021 [33], Canada	Ontario’s vaccine safety surveillance system	Age group [18–64] AND 65 +	Most were females	About 4 (28.6%) reported either a history of allergies (e.g., food, drugs, other vaccines, other common allergens, etc.)	Five (35.7%) persons had a prior episode of anaphylaxis from prior exposures; including to 1^st^ dose of COVID-19 and non-COVID-19 vaccines, drugs, food, and household products	Pfizer-BioNTech, unknown dose	Median [range], 10.5 [9–19.7]	–	Cases were classified as Brighton Level 1 (9 [64%]) or Level 2 (5 [36%]) ^a^	Not measured	Twelve patients (85.7%) were hospitalized and treated in an ED	13 (92.8)	All patients had recovered or been discharged home. No deaths from anaphylaxis were reported	–	(NOS; 6)	Incidence of vaccine-triggered anaphylaxis per 10^6^ doses = number of anaphylaxis cases [14]/doses administered [737,728] = 19
Ontario Public Health Agency 2021 [33], Canada	Ontario’s vaccine safety surveillance system	Age group [18–64]	-	Patient had a prior allergic reaction to non-COVID-19 vaccine	Patient reported a prior history of anaphylaxis to a non-COVID-19 vaccine	Moderna, unknown dose	11 min	–	Case was classified as Brighton Level 1 (1 [100%]) ^a^	Not measured	Patient was treated in an ED	1 (100)	Patient had recovered or been discharged home. No death from anaphylaxis was reported	–	(NOS; 6)	Incidence of vaccine-triggered anaphylaxis per 10^6^ doses = number of anaphylaxis cases (5)/doses administered [152,876] = 6.5
Park et al. 2021 [34], United States	Case report	34-year-old female HCW	0 (0)	A history of childhood asthma and eczema; and a large hive at the site of injection after receiving influenza vaccine. Patient described a greater than 10-year history of inducible episodes of pruritus, swelling, and hives that were most notable at times of sweating. Patient noted an inability to take warm showers as well as limitations with exercise due to pruritus and hives in what was suspicious for cholinergic urticaria	Patient reported no prior history of anaphylaxis	Pfizer-BioNTech, dose 1	3 min	Patient developed flushing, urticaria on her extremities and face, generalized pruritus, tongue swelling, nausea, light-headedness, racing pulse, and SOB	–	Yes; AND level was not elevated	Patient was treated at the ED with I.M. epinephrine and diphenhydramine	1 (100)	Patient recovered with complete resolution of the suspected adverse drug reaction. No death from anaphylaxis was reported	Yes. [Not reported]; and patient showed no reactions to intradermal PEG, polysorbate 80 and Pfizer-BioNTech vaccine	(NOS; 6)	Serum tryptase level was within reference range (4.7 μg/L and 5.4 μg/L) Cholinergic urticaria was confirmed via exercise provocation challenge guided by a standardized protocol (53) Patient tolerated a second dose of Pfizer-BioNTech vaccine, without premedication, exhibiting only transient, mild nausea and a sense of warmth
Pitlick et al. 2021 [35], United States	Retrospective case reports, multicenter	22-year-old female	0 (0)	Allergic rhinitis	Patient reported no prior history of anaphylaxis	Moderna, dose 1	20 min	Angioedema, wheezing, and throat pruritus	Case was classified as Brighton Level 1^a^	Not measured	Patient was treated in the ED and given antihistamines and steroids	0 (0)	Patient recovered with complete resolution of the suspected adverse drug reaction. No death from anaphylaxis was reported	Yes. [Three weeks after the resolution of clinical symptoms]; and patient showed no reactions to intradermal PEG, polysorbate 80, and polysorbate 20	(NOS; 7)	Second vaccine dose was successfully given to patient [51 days after the 1st dose] with a 30-min observation period. Patient experienced minor lip or tongue tingling only
Restivo et al. 2021 [36], Italy	Case report	30-year-old female HCW	0 (0)	A polyallergic subject with a prior reported urticaria-angioedema episode and multiple other immediate cutaneous reaction elicited by chocolate, honey, some cosmetics, and detergents. The first reaction arose a few hours after the ingestion of a meal containing shrimps. Patient had a suspected sensitization to products containing LTP and PEG. Patient was not allergic to vaccines containing polysorbate	Patient reported no prior history of anaphylaxis	Pfizer-BioNTech, dose 1	5 h after the administration of the vaccine	Patient developed erythematous spots on the face and neck and the feeling of a slurred mouth and hoarseness	–	Yes; AND level was not elevated	Patient was treated in the ED and given 8 mg I.V. dexamethasone, one vial I.V. chlorphenamine maleate, 250 mL I.V. 0.9% NaCl, and conventional oxygen therapy (2 L/min)	0 (0)	Patient recovered with complete resolution of the suspected adverse drug reaction. No death from anaphylaxis was reported	Yes. [Two weeks after the resolution of clinical symptoms]; and SPT in patient showed an old dosage of specific IgE with positivity to house dust mites, peach, and other fruits	(NOS; 6)	Patient reported self-administering prednisone (25 mg, one tablet) 14 h before, one tablet 7 h before and one tablet with an I.M. vial (10 mg) of chlorphenamine maleate 1 h before the administration of the vaccine Tryptase value detected in relation to the patient’s medical history did not suggest a mast cell disease The BAT in the patient’s reported history identified the reactivity towards PEG
Sellaturay et al. 2021 [38], United Kingdom	Case report	52-year-old female	0 (0)	Patient had prior allergies or allergic reactions, including to azithromycin containing PEG 6000; shampoos, conditioners, shower gels containing PEG; and toothpastes and mouthwash containing PEG	Patient had prior history of anaphylaxis to drugs	Pfizer-BioNTech, dose 1	1 min	Patient developed throat constriction, cough and then loss of consciousness. She had a respiratory rate of 30/min, tachycardia of 150/min and oxygen saturation of 85%	–	Yes; AND level was not elevated	Patient was treated at the ED with I.M. epinephrine 0.5 mg, I.V. hydrocortisone 200 mg, chlorphenamine 10 mg, and fluids and oxygen 15 L/min	1 (100)	Patient recovered with complete resolution of the suspected adverse drug reaction. No death from anaphylaxis was reported	Yes. [Not reported]; and SPT in patient showed no reactions to Pfizer-BioNTech vaccine, its other excipients, polysorbate 80 and the AstraZeneca COVID-19 vaccine. However, testing was positive to PEG 4000 at 1% of concentration	(NOS; 5)	PEG allergy was diagnosed as the cause of patient Pfizer-BioNTech vaccine anaphylaxis Tryptase levels measured immediately, 1 h and 3 h after this episode were 5.5, 5.5 and 4 ng/ml (normal range 2–14 ng/ml), respectively
Shimabukuro et al. 2021 [39], United States	National passive surveillance (spontaneous reporting) after immunization captured in the VAERS	Median (IQR), 40 (30–50)	2 (9.5)	Seventeen (81%) of 21 patients with anaphylaxis had a documented history of allergies or allergic reactions, including to drugs or medical products, foods, and insect stings	Seven (33%) had experienced an episode of anaphylaxis in the past, including one after receipt of rabies vaccine and another after receipt of influenza A (H_1_N_1_) vaccine	Pfizer-BioNTech, dose 1	Median [range], 13 [2–150]	The most common symptoms and signs were urticaria, angioedema, rash, and a sense of throat closure	All cases were classified as Brighton Level 1 (10 [47.6%]) or Level 2 (11 [52.4%]) ^a^	Not measured	Four patients (19%) were hospitalized (including 3 in ICU), and 17 (81%) were treated in an ED	19 (90.5)	Twenty (95%) were discharged home or had recovered. No deaths from anaphylaxis were reported	–	(NOS; 8)	CDC physicians evaluated these reports and applied Brighton Collaboration case definition criteria to classify case reports as anaphylaxis or not anaphylaxis Incidence of vaccine-triggered anaphylaxis per 106 doses = number of anaphylaxis cases [21]/doses administered [1,893,360] = 11.1
Shimabukuro et al. 2021 [40], United States	National passive surveillance (spontaneous reporting) after immunization captured in the VAERS	Median (IQR), 39 (27–63)	3 (6.4)	Thirty-six (77%) noted a prior allergies or allergic reactions, including to drugs or medical products, foods, and jellyfish stings and unspecified exposures	Sixteen (34%) noted a prior episode of anaphylaxis from prior exposures; included vaccines (rabies, influenza A [H_1_N_1_], seasonal influenza, unspecified), contrast media (gadolinium-based, iodine-based, unspecified I.V.), unspecified infusions, sulfa drugs, penicillin, prochlorperazine, latex, walnuts, unspecified tree nuts, and jellyfish stings	Pfizer-BioNTech, dose 1 (n = 37), dose 2 (n = 4) AND unknown dose (n = 6)	Median [range], 10 [< 1–1140] ^b^	Common symptoms and signs were generalized urticaria, diffuse erythematous rash, angioedema, respiratory and airway obstruction symptoms, and nausea	Cases were classified as Brighton Level 1 (21 [45%]) or Level 2 (23 [49%]) or Level 3 (3 [6%]) ^a^	Not measured	All patients were treated in health care settings; some were treated in an ED and some were hospitalized (including some in ICU, few of whom required ETI)	Most of the anaphylaxis cases (≈92%) received epinephrine as part of emergency treatment	Most of the cases (≈92%) were discharged home or had recovered. No deaths from anaphylaxis were reported	–	(NOS; 8)	CDC physicians evaluated these reports and applied Brighton Collaboration case definition criteria to classify case reports as anaphylaxis or not anaphylaxis Incidence of vaccine-triggered anaphylaxis per 10^6^ doses = number of anaphylaxis cases [47]/doses administered [9,943,247] = 4.7
Shimabukuro et al. 2021 [40], United States	National passive surveillance (spontaneous reporting) after immunization captured in the VAERS	Median (IQR), 41 (24–63)	0 (0)	Sixteen (84%) noted a prior allergies or allergic reactions; including to drugs or medical products, foods, and jellyfish stings and unspecified exposures	Five (26%) noted a prior episode of anaphylaxis from prior exposures; including to drugs or medical products, foods, and jellyfish stings and unspecified exposures	Moderna, dose 1 (n = 17), dose 2 (n = 1) AND unknown dose (n = 1)	Median [range], 10 [1–45]	Common symptoms and signs were generalized urticaria, diffuse erythematous rash, angioedema, respiratory and airway obstruction symptoms, and nausea	Cases were classified as Brighton Level 1 (10 [52%]) or Level 2 (8 [43%]) or Level 3 (1 [5%]) ^a^	Not measured	All patients were treated in health care settings; some were treated in an ED and some were hospitalized (including some in ICU, few of whom required ETI)	Most of the anaphylaxis cases (≈92%) received epinephrine as part of emergency treatment	Most of the cases (≈92%) were discharged home or had recovered. No deaths from anaphylaxis were reported	–	(NOS; 8)	CDC physicians evaluated these reports and applied Brighton Collaboration case definition criteria to classify case reports as anaphylaxis or not anaphylaxis Incidence of vaccine-triggered anaphylaxis per 106 doses = number of anaphylaxis cases [19]/doses administered [7,581,429] = 2.5
Shimabukuro et al. 2021 [39], United States	National passive surveillance (spontaneous reporting) after immunization captured in the VAERS	Median (IQR), 47 (31–63)	0 (0)	Nine (90%) persons had a documented history of allergies or allergic reactions; including to drugs (n = 6), contrast media (n = 2), and foods (n = 1)	Five (50%) persons had a previous history of anaphylaxis; none of which was associated with receipt of a vaccine	Moderna, dose 1	Median [range], 7.5 [1–45]	Vomiting, nausea, respiratory failure, periorbital edema, hypotension, wheezing, erythematous rash, and tongue swelling	Cases were classified as Brighton Level 1 (6 [60%]) or Level 2 (3 [30%]) or Level 3 (1 [10%]) ^a^	Not measured	Six patients were hospitalized (including five in ICU, four of whom required ETI), and four were treated in an ED	10 (100)	All patients had recovered or been discharged home. No deaths from anaphylaxis were reported	–	(NOS; 6)	CDC physicians evaluated these reports and applied Brighton Collaboration case definition criteria to classify case reports as anaphylactic or nonanaphylactic Incidence of vaccine-triggered anaphylaxis per 106 doses = number of anaphylaxis cases [10]/doses administered [4,041,396] = 2.5

*BAT* Basophil Activation Test, *CDC* Centres for Disease Control and Prevention, *CI* confidence interval, *COVID-19* coronavirus disease 2019, *ED* emergency department, *EHRs* electronic health records, *ETI* endotracheal intubation, *h* hour, *HCWs* healthcare workers; ICU, intensive care unit, *IDT* intradermal test, *I.M.* intramuscular, *I.V.* intravenous, *LTP* lipid transfer protein, *NIAID/FAAN* National Institute of Allergy and Infectious Diseases/Food Allergy and Anaphylaxis Network criteria, *NOS* Newcastle Ottawa Scale, *PEG* polyethylene glycol, *RoB 2* Version 2 of the Cochrane risk-of-bias tool for randomized trials, *RR* risk ratio, *SARS-CoV-2* severe acute respiratory syndrome coronavirus 2, *SOB* shortness of breath, *SPT* skin prick test, *VAERS* Vaccine Adverse Event Reporting System

^a^The Brighton Collaboration case definition uses combinations of symptoms to define levels of diagnostic certainty. Brighton level 1 represents the highest level of diagnostic certainty that a reported case is indeed a case of anaphylaxis; levels 2 and 3 are successively lower levels of diagnostic certainty. Level 4 is a case reported as anaphylaxis but that does not meet the Brighton Collaboration case definition. Level 5 is a case that was neither reported as anaphylaxis nor meets the case definition [42]

^b^Time to symptom onset missing in 2 Pfizer-BioNTech reports

^c^NIAID/FAAN clinical criteria for the diagnosis of anaphylaxis must meet 1 of the following criteria: (1) acute onset with involvement of skin and/or mucosal tissue and either (a) respiratory compromise or (b) reduced blood pressure or associated symptoms of end organ dysfunction; (2) 2 or more of the following occur after exposure to a likely allergen for that patient: (a) involvement of skin or mucosal tissue, (b) respiratory compromise, (c) reduced blood pressure or associated symptoms, or (d) persistent gastrointestinal symptoms; and (3) reduced blood pressure after exposure to a known allergen for that patient [43]

^§^An elevated tryptase level was defined as either above the upper limit of normal or > (2 + 1.2 × baseline tryptase level)

**Table 2:** Summary of the characteristics of the included studies with evidence on nonanaphylactic reactions and SARS-CoV-2 vaccines (n = 17), 2020–2021

Author, year, study location	Study design, setting	Age (years)	Male, n (%)	Past history of allergies or allergic reactions	Past history of anaphylaxis	Vaccine brand and dose	Reaction onset after vaccination [min]	Signs and symptoms	Treatment setting: therapy given for reaction	Epinephrine received, n (%)	Outcome	Skin test performed? [Time from reaction to the skin test]; and result	Assessment of study risk of bias (tool used; finding)	Key findings
Ackerman et al. 2021 [18], France	Case report	55-year-old male HCW	1 (100)	No past medical history and no drug allergy	Patient reported no prior history of anaphylaxis	Pfizer-BioNTech, dose 1	180 min	Patient experienced injection-site soreness in the deltoid region with localized pruritic erythematous eruption which later spread on the face, trunk, upper extremities and thighs	Patient was hospitalized and treated with dermocorticoids	Not reported	A gradual improvement over the days with treatment in parallel with the improvement of liver enzymes	Not reported	(NOS; 6)	A persistent maculopapular eruption (1 month) with liver damage [slight hepatic cytolysis (ASAT and GGT 2 N) and biopsy shown haematoxylin and eosin-stained sections showed slight lymphocytic perivascular infiltrate] which led not to give the second dose of Pfizer-BioNTech vaccine
Baden et al. 2021 [19], United States	A phase 3 randomized, observer-blinded, placebo-controlled trial, multicenter	Mean (range), 51.4 (18–95)	7,923 (52.2)	Not reported	Not reported	Moderna, dose 1 and dose 2	Unsolicited delayed adverse events of hypersensitivity (defined in that trial as those with an onset on or after day 8)	Allergic and atopic dermatitis (n = 8), contact dermatitis (n = 21), hypersensitivity (n = 5), injection site rash (n = 37), injection site urticaria (n = 15), rash (n = 45), allergic rhinitis (n = 10), and urticaria (n = 27)	–	–	The reactions typically resolved over the following 4–5 days	–	RoB 2; low risk of bias	Incidence of hypersensitivity reactions per 10^6^ doses = number of nonanaphylactic cases [233]/doses administered [15,185] = 15,344 Moderate, transient reactogenicity after vaccination occurred more frequently in the mRNA-1273 group
Bae et al. 2021 [20], South Korea	A self-administered online questionnaire to HCWs after vaccination	About 190 (68.6%) of the HCWs were in the age group of 20–39	92 (33.2)	Not reported	Not reported	Pfizer-BioNTech, dose 1	Not reported	The most common nonanaphylactic reactions were foreign sensation in the throat (n = 27), throat swelling and tightness (n = 16), nasal obstruction (n = 16), angioedema (n = 12), tongue edema (n = 10), hoarseness (n = 6), skin rash (n = 5), and urticaria (n = 2)	Antipyretics use was less common in the Pfizer-BioNTech vaccine group compared to the AstraZeneca vaccine group regardless of the day of reporting [34.3% vs 82.3%]	–	–	–	(NOS; 5)	Incidence of allergy-like reactions per 10^6^ doses = number of nonanaphylactic cases [94]/doses administered [277] = 339,350 Allergy-like reactions (e.g., foreign body sensation in the throat, swelling in the throat) were significantly less commonly reported in the Pfizer-BioNTech vaccine group compared to the AstraZeneca vaccine group (all p < 0.001)
Bianchi et al. 2021 [21], Italy	Case series	Median [range], 37.5 [27–55]	1 (16.7)	Allergic rhinitis (n = 6), asthma (n = 1), atopic dermatitis (n = 1) and contact allergy to nickel sulphate and fragrances (n = 1)	Patients reported no prior history of anaphylaxis	Pfizer-BioNTech, dose 1	Median (range), 15 (5–1,440 [24 h])	Generalized acute urticaria, angioedema (tongue, gums, and lips), flushing of the face	One case was treated with I.V. betamethasone sodium phosphate	–	–	Yes. [ Not reported]; SPT resulted always negative, but IDT induced, 12 h after, an erythematosus, edematous and infiltrated asymptomatic reaction in all patients	(NOS; 6)	Incidence of mucous-cutaneous adverse reactions per 10^6^ doses = number of nonanaphylactic cases [6]/doses administered [5574] = 1076 All patients then received the second dose of vaccine without relapses
Blumenthal et al. 2021 [22], United States	Case series	Median (IQR), 44 (37.2–48.5)	2 (16.7)	About 8 (66.7%) of the cases had prior contrast allergy (hives), penicillin, sulfasalazine and influenza vaccine allergy (hives and fever), urticaria, wasp allergy (hives), almond allergy (hives), angioedema, rhinitis, and rash. However, four (33.3%) cases had no allergy history	Patients reported no prior history of anaphylaxis	Moderna, dose 1	Delayed adverse events of hypersensitivity (defined as those with an onset on or after day 8); and a range of 4–11 days	Pruritus, burning, pain, warmth, erythema, induration, swelling and hyperpigmentation. Five of the reactions were Grade 3 plaques (≥ 10 cm in diameter)	Most patients received treatment for their symptoms (e.g., with ice and antihistamines). Some patients received glucocorticoids, and 1 patient received antibiotic therapy for presumptive cellulitis	–	The symptoms resolved a median of 6 days after onset (range, 2 to 11)	–	(NOS; 7)	All 12 patients completed Moderna vaccination. Although half the patients did not have a recurrence of large local reactions, 3 patients had recurrent reactions that were similar to those after the initial dose, and 3 patients had recurrent reactions that were of a lower grade than those after the initial dose. The median onset of cutaneous symptoms after the 2nd dose (day 2; range, 1–3) was earlier than that after the 1st dose Second vaccine dose was administered with premedication [including cetirizine, diphenhydramine, fexofenadine and loratadine] in 8/12 [66.7%] of the cases Authors also reported a 27-year-old Caucasian male who developed an erythematous plaque at the site of Moderna vaccine inoculation, 7 days after injection of Dose 1. A skin punch biopsy was taken and was found to represent a T-cell–mediated hypersensitivity reaction
CDC COVID-19 Response Team 2021 [24], United States	National passive surveillance (spontaneous reporting) after immunization captured in the VAERS	Median [range], 43 [18–65]	8 (9.6)	For 56/83 (67%) case reports, a past history of allergies or allergic reactions, mostly to foods and drugs, was documented	Patients reported no prior history of anaphylaxis	Pfizer-BioNTech, dose 1	12 (< 1–1,200 [20 h])	Commonly symptoms included pruritus, rash, itchy and scratchy sensations in the throat, and mild respiratory symptoms	Not reported	Not reported	All patients had recovered or been discharged home. No deaths from anaphylaxis were reported	–	(NOS; 7)	Incidence of nonanaphylactic reactions per 10^6^ doses = number of nonanaphylactic cases [83]/doses administered [1,893,360] ≈ 4 CDC physicians evaluated these reports and applied Brighton Collaboration case definition criteria to classify case reports as anaphylaxis or not anaphylaxis About 72/83 (87%) of the nonanaphylactic allergic reactions were classified as nonserious
Corbeddu et al. 2021 [23], Italy	Case series	Median [range], 56 [36–61]	4 (36.4)	Majority of patients (72.7%, n = 8) had a previous history of allergy or allergic diathesis	Patients reported no prior history of anaphylaxis	Pfizer-BioNTech, dose 1 AND dose 2	Median (range), 48 h (1 h–72 h)	Cutaneous symptoms [such as erythematoedematous reaction at injection site, diffuse morbilliform rash, mild erythema and positive dermographism]	The patient who manifested a relapse of atopic dermatitis underwent a short oral steroids course prescribed by his GP	–	All manifestations resolved spontaneously within 2–3 days without treatment, except in the patients with extracutaneous symptoms	–	(NOS; 6)	Incidence of cutaneous symptoms per 106 doses = number of nonanaphylactic cases (5)/doses administered [3170] = 3470 Authors concluded cutaneous reactions observed in their series were very mild and do not constitute a contraindication to a 2nd dose of vaccine
Johnston et al. 2021 [26], United States	Case series	Median [range], 38 [25–89]	3 (18.7)	About 1/16 (6.2%) reported a prior localized vaccine reaction (mild reaction to an influenza vaccine)	Patients reported no prior history of anaphylaxis	Moderna, dose 1 AND dose 2	Median [range], 7 days [2–12 days]	HCWs experienced delayed localized cutaneous reactions that occurred at or near the injection site and were described as pruritic, painful, and edematous pink plaques	Treatments included topical steroids, oral antihistamines, and cool compresses; 1 patient had received cephalexin for presumed cellulitis	–	–	–	(NOS; 8)	Reactions to the first vaccine dose had a median (range) duration of 5 (1–21) days Skin biopsy specimen demonstrated a mild predominantly perivascular mixed infiltrate with lymphocytes and eosinophils, consistent with a dermal hypersensitivity reaction Of participants who had a reaction to first vaccine dose (15 of 16 patients), most (11 patients) developed a similar localized injection-site reaction to the second vaccine dose; most (10 patients) also developed the second reaction sooner as compared with the first-dose reaction
Kadali et al. 2021 [27], United States	A randomized, cross‐sectional study using an independent online survey questionnaire was conducted to collect responses from HCWs	Average age of all participants who completed the survey (n = 432) was 43.76 years	About 10.65% of participants were males	Not reported	Not reported	Moderna, dose 1 AND dose 2	Not reported	Among the 432 vaccine recipients, (n = 58) reported rash*, (n = 35) reported palpitations, (n = 15) reported cough, (n = 10) reported SOB, (n = 8) reported chest pain, (n = 7) reported hives, (n = 4) reported syncope, and (n = 2) reported swelling in the mouth/throat	Only 17/432 (3.94%) required seeking help from an outpatient provider, 1/432 (0.23%) required seeking help from ED providers, and none of the participants required hospitalization	–	Patients recovered with complete resolution of the suspected adverse drug reactions. No deaths were reported	–	RoB 2; low risk of bias	Incidence of nonanaphylactic reactions per 10^6^ doses = number of nonanaphylactic cases [139]/doses administered [432] = 321,759 Most of the symptoms reported were nonlife threatening. Despite the wide array of self-reported symptoms, there appears to be a higher acceptance for this vaccine
Kelso et al. 2021 [28], United States	Case reports	Median (IQR), 45 (42.2–53.7)	0 (0)	Not reported	Not reported	Pfizer-BioNTech (n = 3) AND Moderna (n = 1); both brands after dose 1	Median [range], 3.5 [1.2–12.5]	Sensation of throat swelling, SOB, hot and sweaty, headache, disoriented, pruritus, itching, flushing, puffy eyelids, and hives	One patient (25%) was hospitalized and needed ICU healthcare and other 3 patients (75%) were treated at the ED; and the 4 cases patients were treated with prednisone, cetirizine, epinephrine, famotidine, methylprednisolone, diphenhydramine, dexamethasone, hydroxyzine, epinephrine and diazepam	2 [50]	Patients recovered with complete resolution of the suspected adverse drug reactions. No deaths were reported	Yes. [ Not reported]; and SPTs and IDTs in all patients showed no reactions to the individual COVID-19 vaccine taken previously as a 1^st^ dose	(NOS; 6)	Subsequent vaccine skin testing suggested that the reactions were not IgE-mediated [anaphylactic], and 3 of 4 have received their second doses either without symptoms or with only mild transient symptoms Despite negative skin testing results, 1 patient declined her second dose. The skin test results and subsequent vaccination outcomes in these patients indicate that their initial reactions were not allergic
McMahon et al. 2021 [30], United States	An international registry of COVID-19 vaccine cutaneous reactions by HCWs only	Median (IQR), 45 (36–60)	29 (8.4)	Among the 343 cases, (n = 12) reported atopic dermatitis, (n = 10) reported contact dermatitis, (n = 6) reported psoriasis, (n = 5) reported urticaria and (n = 4) reported acne vulgaris	Not reported	Moderna, dose 1 AND dose 2	First dose: Median [IQR], 7 days [2–8] Second dose: Median [IQR], 1 days [1, 2]	Among the 369 administered doses [1^st^ and 2^nd^], there were nausea (n = 43), urticaria (n = 23), morbilliform (n = 18), diarrhea (n = 13), angioedema (n = 5), and erythema multiforme (n = 3)	Patients responded well to topical corticosteroids, oral antihistamines, and/or pain-relieving medications. Antibiotics were not required for resolution but were sometimes given by HCWs concerned that the reaction might be cellulitis	Not reported	No patients with these findings experienced anaphylaxis or another severe adverse event. These reactions resolved after a median of 3–4 days	Not reported	(NOS; 6)	Incidence of nonanaphylactic reactions per 10^6^ doses = number of nonanaphylactic cases [105]/doses administered [369] ≈ 284,553 For both Moderna and Pfizer-BioNTech vaccines, cutaneous reactions were reported mostly by dermatologists (30%), other physicians (26%), and other HCWs (22%); and almost all the cases came from the United States (98%) About 414 cutaneous reactions to mRNA COVID-19 vaccines were recorded from Moderna (83%) and Pfizer-BioNTech (17%) For both vaccines, about 21% reported reactions after the 1st dose only, 63% reported a reaction after the 2nd dose only, and 16% reported reactions to both doses
McMahon et al. 2021 [30], United States	An international registry of COVID-19 vaccine cutaneous reactions by HCWs only	Median (IQR), 42 (36–54)	11 (15.5)	Among the 71 cases, (n = 5) reported atopic dermatitis, (n = 2) reported contact dermatitis, (n = 3) reported psoriasis, (n = 2) reported urticaria and (n = 2) reported acne vulgaris	Not reported	Pfizer-BioNTech, dose 1 AND dose 2	First dose: Median [IQR], 7 days [2–8] Second dose: Median [IQR], 1 days [1, 2]	Among the 74 administered doses [1^st^ and 2^nd^], there were urticaria (n = 17), morbilliform (n = 9), nausea (n = 7), angioedema (n = 1), and diarrhea (n = 1)	Patients responded well to topical corticosteroids, oral antihistamines, and/or pain-relieving medications. Antibiotics were not required for resolution but were sometimes given by HCWs concerned that the reaction might be cellulitis	Not reported	No patients with these findings experienced anaphylaxis or another severe adverse event. These reactions resolved after a median of 3–4 days	Not reported	(NOS; 6)	Incidence of nonanaphylactic reactions per 10^6^ doses = number of nonanaphylactic cases [35]/doses administered [74] ≈ 472,973 Delayed large local reactions were most common, followed by local injection site reactions, urticarial eruptions, and morbilliform eruptions Additional less common reactions included pernio/chilblains, cosmetic filler reactions, zoster, herpes simplex flares, and pityriasis rosea-like reactions Serious adverse events did not develop in any of the patients in the registry after the first or second dose
Mustafa et al. 2021 [31], United States	Two case reports	A 64-year-old female (patient 1) AND a 39-year-old female (patient 2)	0 (0)	Patient 1 had history of shellfish allergy AND patient 2 had a history of allergic rhinitis	Both patients reported no prior history of anaphylaxis	Moderna, dose 1	10 min (patient 1) AND 15 min (patient 2)	Patient 1 had generalized pruritus, urticaria, and self-reported tachycardia but no angioedema, respiratory or gastrointestinal symptoms, or hypotension Patient 2 developed chest neck urticaria and mild facial angioedema	Patient 1 was treated at vaccination site and given 50 mg of oral diphenhydramine Patient 2 was given 25 mg of oral diphenhydramine at the vaccination site, then transported by ambulance to the ED, where she received 20 mg of I.V. famotidine and 125 mg of methylprednisolone	0 (0)	Both patients recovered with complete resolution of the suspected adverse drug reaction	Yes. [Not reported]; and SPTs in both patients showed no reactions to PEG, polysorbate, and Moderna; however, IDTs in the two patients shown positive reaction to Moderna	(NOS; 5)	Second vaccine dose was administered without premedication through a graded dosing protocol. Patient 1 had no symptoms during the protocol. Patient 2 reported pruritus after doses 2 and 5, but it resolved without medical intervention. Both patients reported no additional symptoms over the following 24 h In addition, 3 to 4 weeks after receiving the second dose, both patients had IgG antibodies directed against the spike protein of COVID-19, suggesting vaccination was efficacious
Ocáriz et al. 2021 [32], Spain	Investigational clinical trial, single centre	43-year-old female	0 (0)	A history of severe asthma	Patient reported no prior history of anaphylaxis	Pfizer-BioNTech, dose 1	10 min	Patient developed nasal obstruction and rhinolalia and pruriginous erythematous macules on the neck and upper thorax	Patient was treated at the allergy department and given I.M. dexchlorpheniramine	0 (0)	Patient recovered with complete resolution of the suspected adverse drug reaction	Yes. [Not reported]; and SPT in patient was not allergic to PEG-3350	RoB 2; some concerns risk of bias	No modification on tryptase levels compared with the baseline was found
Pitlick et al. 2021 [35], United States	Retrospective case reports, multicenter	Median (IQR), 36 (24–52)	2 (28.6)	About 4 (57.1%) of the cases had prior drug (influenza vaccine and antihistamines) and food allergy; and asthma. However, two (28.6%) cases had no allergy history	Two (28.6%) cases had prior insect stinging (venom) anaphylaxis	Pfizer-BioNTech (n = 6) AND Moderna (n = 1); both brands after dose 1	Median [range], 97 [6–405]	Urticaria, tachycardia, rhinorrhea, facial flushing, oral pruritus, throat fullness and tightness, and angioedema	Patients were treated at the vaccination sites and given antihistamines and steroids	–	Patient recovered with complete resolution of the suspected adverse drug reaction. The symptoms resolved a median of 6 h after onset (range, 0.2 to 24)	Yes. [A median of 16 days after the resolution of clinical symptoms (range, 9 to 20); and patients showed no reactions to PEG, polysorbate 80, and polysorbate 20	(NOS; 7)	All patients successfully received their 2nd vaccine dose without premedication or split-dosing. Patients experienced no allergic reactions
Riad et al. 2021 [37], The Czech Republic	A cross-sectional self-administered online questionnaire	About 50.2% of participant were ≤ 43 years old	100 (11.4)	About 21.8% and 5.9% of participants reported a history of asthma and allergy, respectively	Patients reported no prior history of anaphylaxis	Pfizer-BioNTech, dose 1 AND dose 2	Not reported	Rash (n = 28), urticaria (n = 10), tongue tingling (n = 5), and swollen lips (n = 4)	–	–	–	–	(NOS; 6)	Incidence of nonanaphylactic reactions per 106 doses = number of nonanaphylactic cases [47]/doses administered [877] ≈ 53,592 Rash and urticaria were more common among the ≤ 43 years old group than the older age group
Shimabukuro et al. 2021 [39], United States	National passive surveillance (spontaneous reporting) after immunization captured in the VAERS	Median [range], 43 [22–96]	4 (9.3)	For 26/43 (60%) case reports, a past history of allergies or allergic reactions, mostly to foods and drugs, was documented	Patients reported no prior history of anaphylaxis	Moderna, dose 1	15 (< 1–1,440 [24 h])	Commonly symptoms included pruritus, rash, itchy sensations in the mouth and throat, sensations of throat closure, and respiratory symptoms	–	–	All patients had recovered or been discharged home. No deaths from anaphylaxis were reported	-	(NOS; 6)	Incidence of vaccine-triggered anaphylaxis per 106 doses = number of anaphylaxis cases [43]/doses administered [4,041,396] = 10.6 CDC physicians evaluated these reports and applied Brighton Collaboration case definition criteria to classify case reports as anaphylactic or nonanaphylactic About 26/43 (60%) of the nonanaphylactic allergic reactions were classified as nonserious
Vieira et al. 2021 [41], Portugal	Prospective cohort, single center	31	0 (0)	Allergic rhinitis	Sixty reported a previous history of drug anaphylaxis	Pfizer-BioNTech, dose 1	10	HCW developed a sudden onset generalized urticaria	–	–	–	A total of 115 HCWs performed SPTs and IDTs with the Pfizer-BioNTech SARS-CoV-2 vaccine (83% women, mean age of 44 ± 12 years)	(NOS; 6)	SPTs and IDTs were performed for each person with the undiluted vaccine and a 1/10 dilution with saline solution Fifty-five of participants were control subjects, who had tolerated the vaccine Of the 60 participants who reported a history of anaphylaxis, four subjects had positive results to the IDTs. All 4 were then tested with PEG 2000 with SPTs and IDTs. Only one tested positive to the IDTs A HCW developed generalized urticaria was to Pfizer-BioNTech was premedicated with 20 mg of cetirizine

*CDC* Centres for Disease Control and Prevention, *COVID-19* coronavirus disease 2019, *ED* emergency department, *GP* general practitioner, *h* hour, *HCWs* healthcare workers, *ICU* intensive care unit, *IDT* intradermal test, *I.M.* intramuscular, *I.V.* intravenous, *NOS* Newcastle Ottawa Scale, *PEG* polyethylene glycol, *RoB 2* Version 2 of the Cochrane risk-of-bias tool for randomized trials, *SARS-CoV-2* severe acute respiratory syndrome coronavirus 2, *SOB* shortness of breath, *SPT* skin prick test, *VAERS* Vaccine Adverse Event Reporting System

*Rash was reported as both localized side effect and allergic side effect

The following data were extracted from selected studies: authors; publication year; study location; study design and setting; age; proportion of male patients; past history of allergies or allergic reactions and/or anaphylaxis; vaccine brand and dose [if 1st dose, 2nd dose or both]; time from vaccination to anaphylactic or nonanaphylactic reaction onset; signs and symptoms; anaphylaxis level of severity if occurred; treatment setting and therapy received for reaction; if epinephrine was received; treatment outcome; if skin test was performed and test result; assessment of included study risk of bias (tool used; finding) and; remarks on notable findings.

### Quality assessment

The quality assessment of the studies was undertaken mainly based on the Newcastle–Ottawa Scale [NOS] to assess the quality of the selected studies [12]. This assessment scale has two different tools for evaluating case–control and cohort studies. Each tool measures quality in the three parameters of selection, comparability, and exposure/outcome, and allocates a maximum of 4, 2, and 3 points, respectively. High-quality studies are scored greater than 7 on this scale, and moderate-quality studies, between 5 and 7. Revised Cochrane risk of bias tool (RoB 2.0) was used to assess the risk of bias in randomized controlled studies [13]. Quality assessment was performed by five authors (H.M.A., A.S.A., T.T.A., G.A., and A.M.A.) independently, with any disagreement to be resolved by consensus.

### Data analysis

The primary outcome of interest was to determine the incidence rates of anaphylactic and nonanaphylactic reactions induced after the administration of mRNA COVID-19 vaccines [namely Pfizer-BioNTech and Moderna]. Secondary outcome was to analyze identified evidence describing the demographic and clinical characteristics, triggers, presenting signs and symptoms, treatment and clinical course of confirmed cases with anaphylactic and nonanaphylactic reactions induced by those two mRNA vaccines. Taking a conservative approach, a random effects meta-analysis with the Hunter-Schmidt model was used [14], which produces wider confidence intervals [CIs] than a fixed effect model. Results were illustrated using forest plots. Statistical heterogeneity was evaluated using the Cochran’s chi-square (*χ*^*2*^) and the *I*^*2*^ statistic [15]. An *I*^*2*^ value of > 50% is suggestive of significant heterogeneity [16]. To detect the source of heterogeneity, subgroup analysis was performed based on vaccine brand administered [Pfizer-BioNTech or Moderna]. Publication bias was evaluated using funnel plots and the Egger’s correlation test, with p < 0.05 indicating statistical significance [17]. All analyses were done using R version 4.1.0 with the packages metafor and meta.

## Results

### Study characteristics and quality

**Fig. 1:**
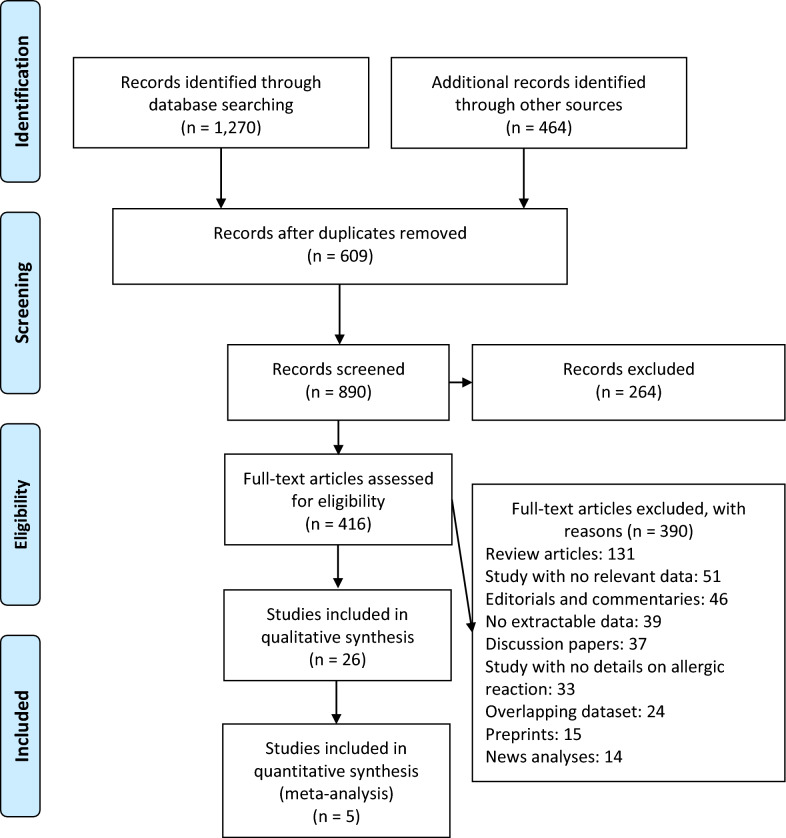
Flow diagram of literature search and data extraction from of studies included in the systematic review and meta-analysis

A total of 1,734 publications were identified (Fig. 1). After scanning titles and abstracts, we discarded 609 duplicate articles. Another 264 irrelevant articles were excluded based on the titles and abstracts. The full texts of the 416 remaining articles were reviewed, and 390 irrelevant articles were excluded. As a result, we identified 26 studies that met our inclusion criteria [18–41]. The detailed characteristics of the included studies are shown in Tables 1 and 2. Among the included studies, 12 reported anaphylaxis reactions [22, 25, 27, 29, 33–36, 38–40], 17 reported nonanaphylactic reactions [18–24, 26–28, 30–32, 35, 37, 39, 41], and 3 studies reported both anaphylactic and nonanaphylactic reactions (27, 35, 40). The majority of studies reporting incidence rates of anaphylaxis and nonanaphylactic reactions were conducted based on registry databases [22, 24, 30, 33, 39, 40], and most were from hospital and/or emergency department admission databases [18, 19, 21–23, 25–28, 30–36, 38–41]. A total of 26,337,421 mRNA SARS-CoV-2 vaccines recipients [Pfizer-BioNTech (n = 14,505,399) and Moderna (n = 11,831,488)] were included in the systematic review and meta-analysis, 89.1% [23] of whom were part of the US-VAERS [24, 30, 39, 40] and Ontario’s vaccine safety surveillance system to report anaphylactic and nonanaphylactic events [33]. Anaphylactic and nonanaphylactic reactions were reported by vaccine recipients via an online self-administered survey [20, 22, 27, 29, 37]; and only few studies in which anaphylactic events received through electronic health records were reviewed by allergists and immunologists [22] or physicians with unspecified medical specialty [39, 40]. Only few nonanaphylactic reactions were reported by dermatologists, other physicians, and other HCWs [30]. There were 8 case report [18, 25, 28, 31, 34–36, 38], 5 cohort [20, 22, 29, 37, 41], 4 case series [21–23, 26], 2 randomized controlled trial [19, 32] and 1 randomized cross-sectional [27] studies. These studies were conducted in United States (n = 15), Italy (n = 3), United Kingdom (n = 2), Canada (n = 1), France (n = 1), South Korea (n = 1), Spain (n = 1), Portugal (n = 1), and Czech Republic (n = 1). Only 3 studies were performed with a multi-centre design [19, 22, 35]. The median NOS score for these studies was 6 (range, 5–7). Among the 26 included studies, 22 studies were moderate-quality studies (i.e., NOS scores were between 5 and 7) [18–25, 27–39, 41] and 4 studies demonstrated a relatively high quality (i.e., NOS scores > 7) [22, 26, 39, 40]; Tables 1 and 2.

Five and three studies exclusively reported anaphylaxis events in vaccine recipients of Pfizer-BioNTech [25, 34, 36, 38, 39] and Moderna [27, 35, 39], respectively; and four studies reported on anaphylactic reactions after the administration of both mRNA vaccines [22, 29, 33, 40]. Nonanaphylactic reactions were reported in patients after receiving Pfizer-BioNTech [18, 20, 21, 23, 24, 32, 37, 41] or Moderna [19, 22, 26, 27, 31, 39]; and few studies reported on nonanaphylactic reactions due to the vaccination by both COVID-19 vaccines [28, 30, 35]. Most studies described anaphylactic and nonanaphylactic reactions after receiving first dose of vaccine [18, 20–22, 24, 25, 31, 32, 34–36, 38, 39, 41]; however, reactions were also reported after the second dose [19, 23, 26–30, 37], and in few studies the given dose was unknown [27, 33, 40].

Essential to exclude the presence of mastocytosis and to characterize the anaphylactic events that might occurred during vaccination, only four studies reported measurement of tryptase levels in patients who suffered anaphylactic reactions (22, 34, 36, 38); Tables 1 and 2.

### Incidence of anaphylactic and nonanaphylactic reactions to mRNA COVID-19 vaccines

Five and six studies offered varying estimates of incidence rates for the occurrence of anaphylaxis to Pfizer-BioNTech [22, 29, 33, 39, 40] and Moderna [22, 27, 29, 33, 39, 40] vaccines; while six and four studies presented varying estimates of incidence rates for the occurrence of nonanaphylactic reactions to Pfizer-BioNTech [20, 21, 23, 24, 30, 37] and Moderna [19, 27, 30, 39] vaccines; respectively, as shown in Tables 1 and 2.

**Fig. 2:**
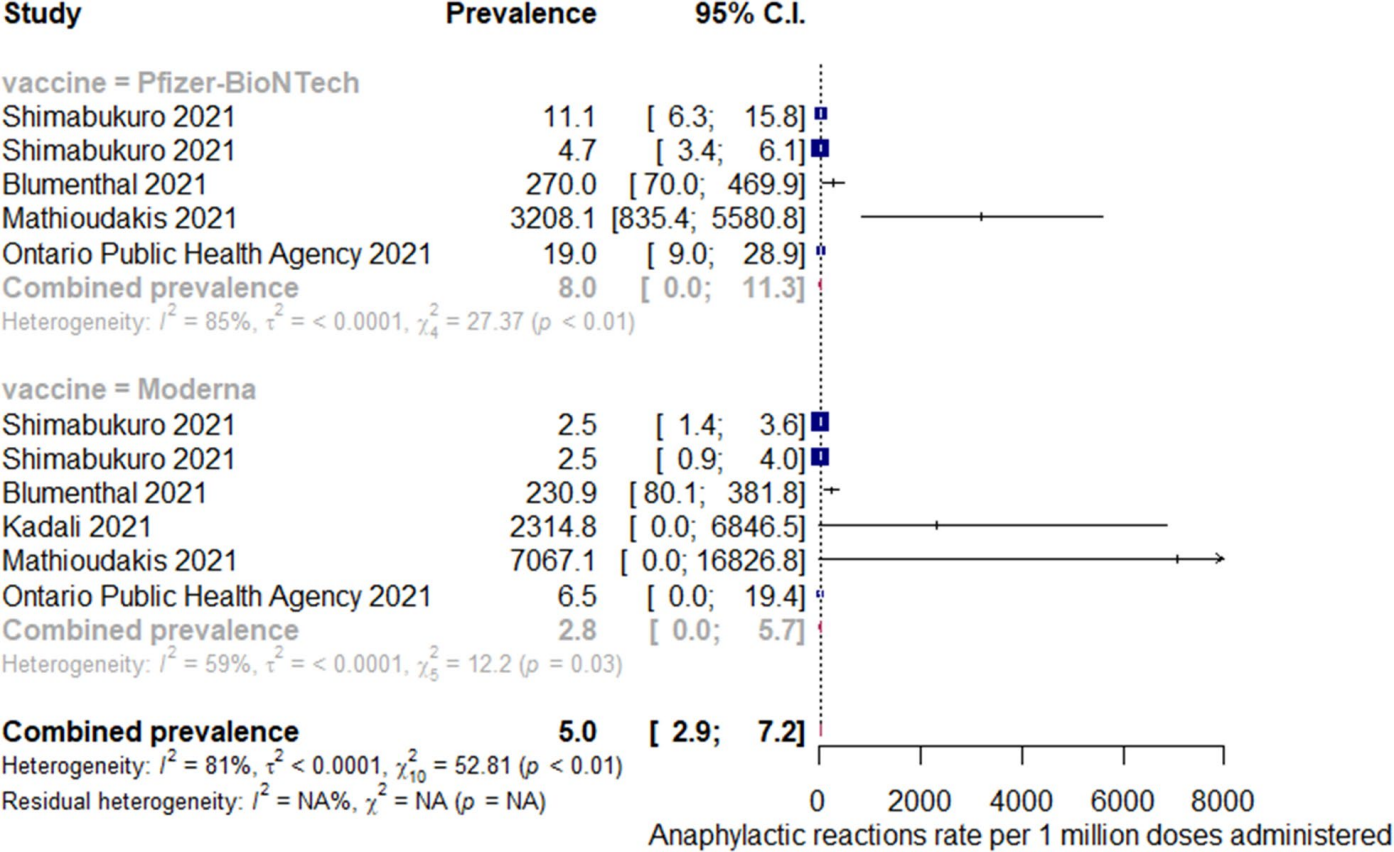
Pooled estimate for the prevalence of anaphylaxis associated with mRNA COVID-19 immunization stratified by the vaccine type

**Fig. 3:**
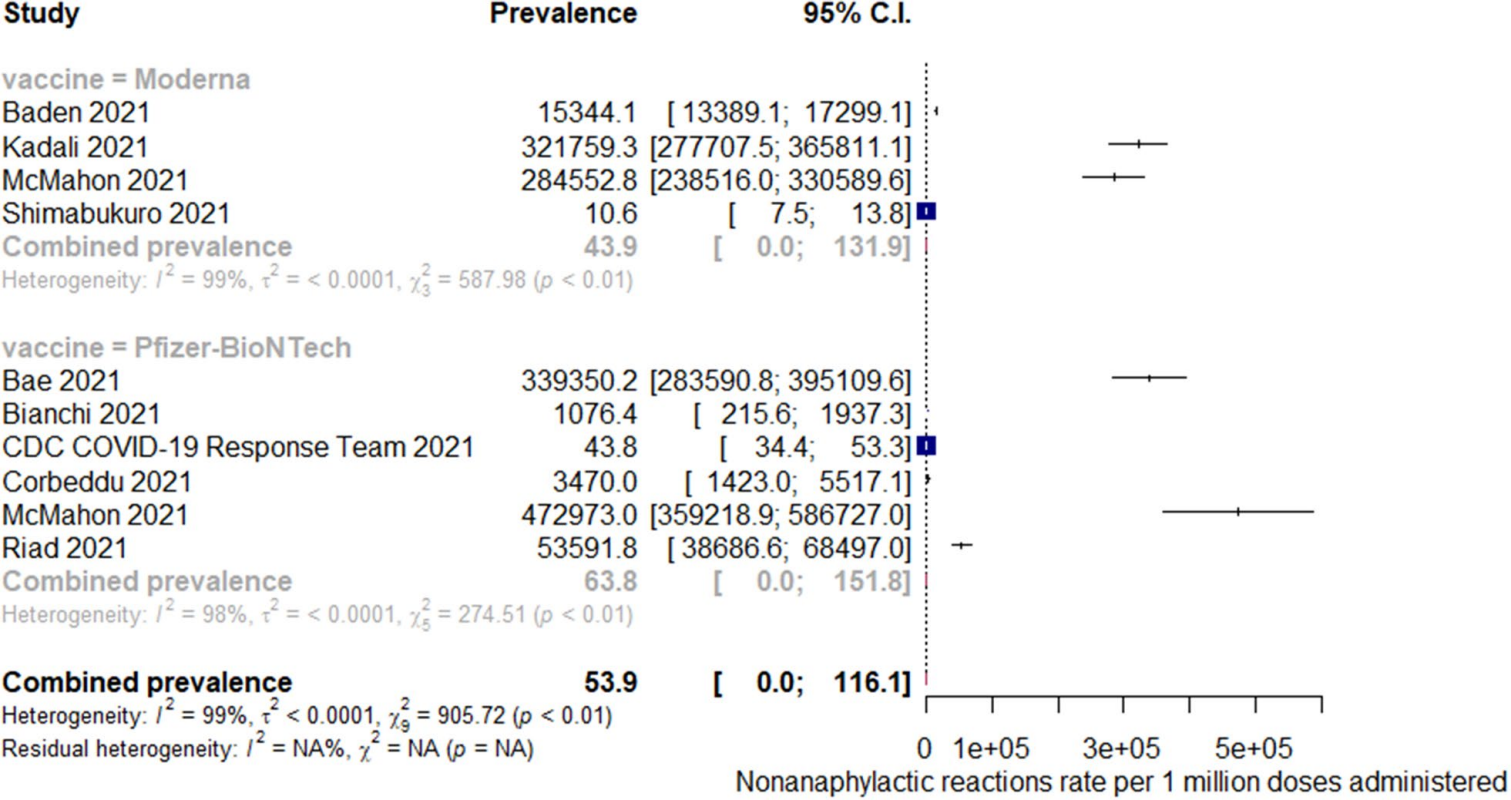
Pooled estimate for the prevalence of nonanaphylactic reactions associated with mRNA COVID-19 immunization stratified by the vaccine type

**Fig. 4:**
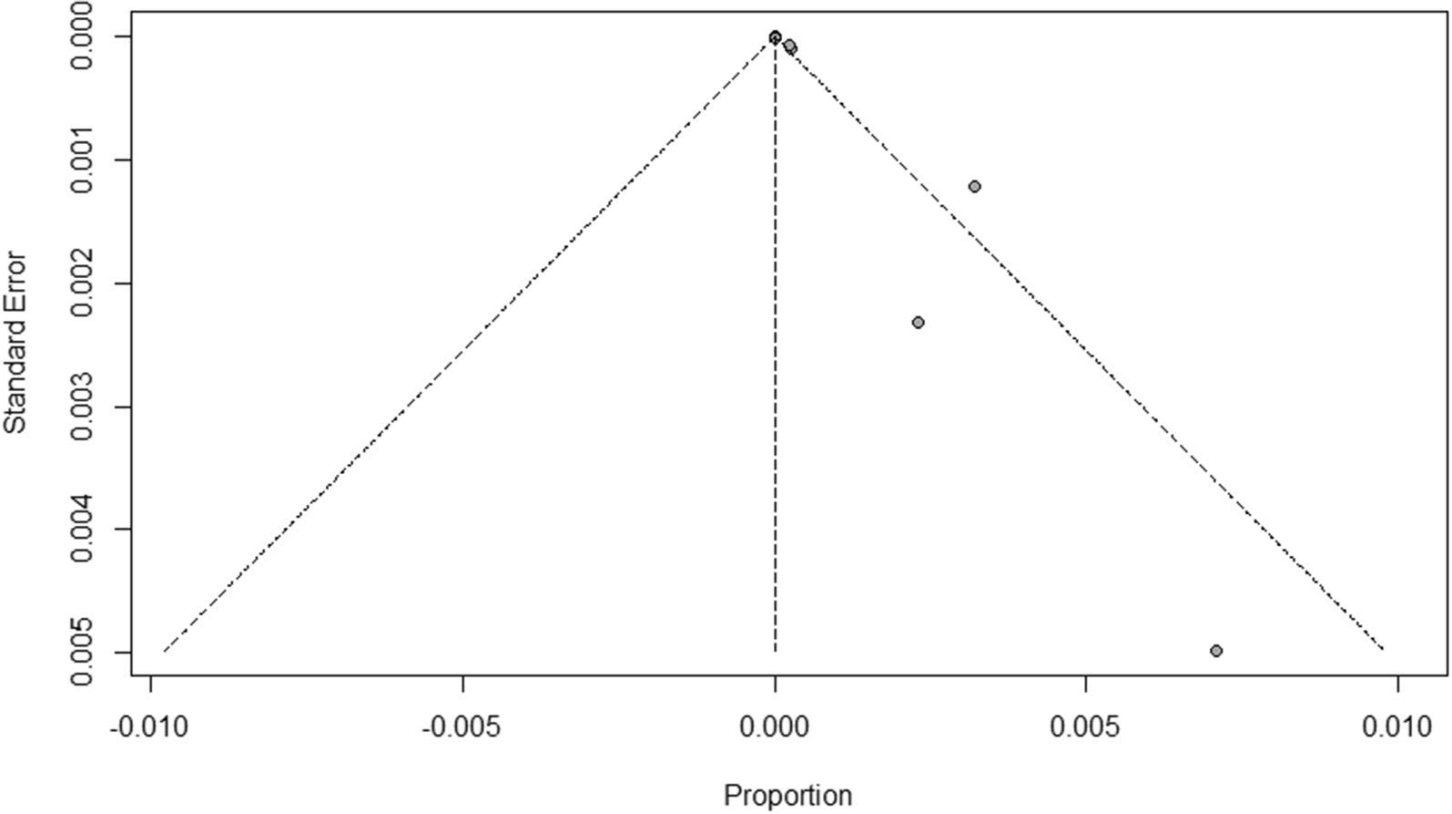
Funnel plots to evaluate publication bias for the pooled effect size to determine the prevalence of anaphylaxis associated with mRNA COVID-19 immunization based on mRNA vaccine type

**Fig. 5:**
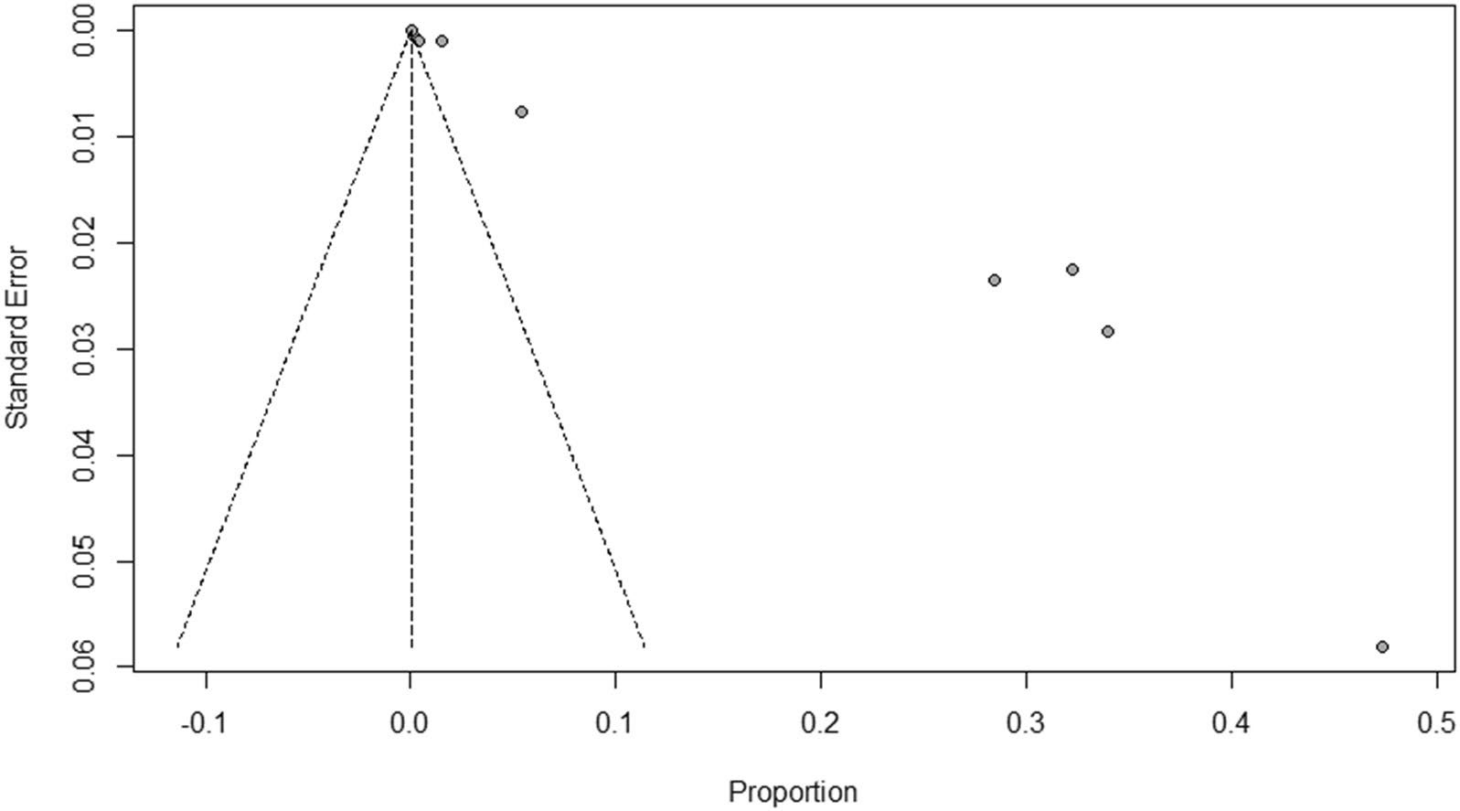
Funnel plots to evaluate publication bias for the pooled effect size to determine the prevalence of nonanaphylactic reactions associated with mRNA COVID-19 immunization based on mRNA vaccine type

The incidence rates for all-cause anaphylaxis to both vaccine brands ranged from 2.5 to 7,067 per one million doses administered [22, 27, 29, 33, 39, 40]; while incidence rates for all-cause nonanaphylactic reactions to both vaccines ranged from 10.6 to 472,973 per one million doses administered [19–21, 23, 24, 27, 30, 37, 39]. The overall pooled prevalence estimate of anaphylaxis to both vaccines was 5.58 (95% CI 3.04–8.12, *I*^*2*^ = 76.32%, *p* = 0.00), while the overall pooled prevalence estimate of nonanaphylactic reactions to both vaccines was 89.53 (95% CI − 11.87–190.94, *I*^*2*^ = 97.08%, *p* = 0.00). Vaccination with Pfizer-BioNTech resulted in higher anaphylactic reactions compared to Moderna (9.31, 95% CI 4.23 to 14.40, *I*^*2*^ = 52.55% versus 3.42, 95% CI 1.42–5.41, *I*^*2*^ = 49.43%), as shown in Fig. 2. However, lower incidence of nonanaphylactic reactions was associated with Pfizer-BioNTech compared to Moderna (75.27, 95% CI − 48.28–198.82, *I*^*2*^ = 0.77% versus 99.01, 95% CI − 49.77–247.80, *I*^*2*^ = 0.37%), as shown in Fig. 3. The funnel plots for possible publication bias for the pooled effect sizes to determine the incidence of anaphylaxis and nonanaphylactic reactions associated with mRNA COVID-19 immunization based on mRNA vaccine type appeared asymmetrical on visual inspection, and Egger’s tests confirmed asymmetry by producing *p* values < 0.05; Figs. 4 and 5.

### Characteristics of the patients, history of allergy, and co-morbid conditions

The median or mean patient age ranged from ≥ 18 to 96 years across studies (i.e., only adult patients were included). There was an increased female predominance in vaccine-associated anaphylactic and nonanaphylactic reactions in most of the studies [20–41]. The key triggers to anaphylactic reactions identified in these studies included foods [22, 25, 33, 36, 39, 40], medications [22, 25, 33, 38–40], stinging insects [39] or jellyfish [40], cosmetics and detergents [36, 38], household products [33], and latex [40]. Nonanaphylactic reactions were triggered less by foods [22, 24, 31, 35, 39], drugs [22, 24, 26, 35, 39, 41], insect stinging [22], and contrast media [22]. Previous history of anaphylaxis [22, 25, 33, 35, 38–41]; and comorbidities such as asthma [21, 32, 34, 35, 37], allergic rhinitis [21, 22, 31, 35, 41], atopic and contact eczema/dermatitis [21, 30, 34] and psoriasis [30] and cholinergic urticaria [34] were also found to be important.

### Reaction onset after vaccination, clinical features and severity of the reaction

Anaphylactic reactions onsets after inoculation by both vaccines were within 1–5 min [34, 38], > 5–30 min [22, 25, 33, 35, 39, 40], > 30 min [36], or not stated in cases [27, 29]; whereas nonanaphylactic reactions occurred within < 5 min [28], > 5–30 min [21, 24, 31, 32, 39, 41], > 31 to ≤ 1,440 min [18, 30, 35], ≥ 2–8 days [19, 22, 23, 26, 30], or not reported in cases [20, 27, 37]. The common presenting signs and symptoms in anaphylactic cases were pruritic hives [22, 25, 34, 39, 40]; throat closure [22, 25, 38, 39] or pruritis [35]; angioedema [22, 35, 39, 40]; wheezing [22, 25, 35, 39]; nausea and vomiting [22, 34, 39, 40]; tachycardia; rash [39, 40]; hypotension [22, 39]; cough [22, 38]; dyspnea [22, 25]; tongue swelling [34, 39]; flushing [34]; diarrhea [22]; light headedness [34]; shortness of breath [34]; respiratory failure [39]; periorbital oedema [39]; loss of consciousness [38]; hypoxemia [38]; and erythematous spots on face and neck, slurred mouth, and hoarseness [36] (Table 1). The definition from Brighton criteria [42] was the most widely applied definition in included studies (n = 7) [22, 25, 33, 35, 39, 40]; although 1 study [22] relied on NIAID/FAAN criteria [43] and Brighton criteria [42] to define anaphylaxis. On severity assessment, most anaphylactic patients belonged to Brighton Level 2 (n = 55) [22, 33, 39, 40]; Brighton Level 1 (n = 50) [22, 25, 33, 35, 39, 40]; National Institute of Allergy and Infectious Diseases/Food Allergy and Anaphylaxis Network [NIAID/FAAN] Grade II (n = 9) [22] or Grade I (n = 7) [22]; and Brighton Level 3 (n = 6) [22, 40] at the time of presentation.

Most frequently-reported nonanaphylactic reactions following COVID-19 vaccines were cutaneous reactions (18–20, 23, 24, 27, 32, 35, 39) and delayed large local reactions (19, 22, 26, 30). Both cutaneous and delayed reactions frequently took the form of injection site swelling and/or pain, erythema, and rash. Another commonly-reported nonanaphylactic reaction was urticaria [21, 22, 28, 30, 31, 37, 41]. These common reactogenicity symptoms occurred in lower rates following the second vaccine dose [22, 26, 30]. There were less frequent and unusual cutaneous reactions to the COVID-19 vaccines such as: erythema multiforme, pernio/chilblains, filler swellings, and exacerbation of viral infections such as herpes zoster and herpes simplex in addition to the occurrence of pityriasis rosea-like reactions (27, 30).

### Therapeutic interventions and treatment outcome

Epinephrine was administered in 99 anaphylactic cases [22, 25, 33, 34, 38–40] and four studies did not state its use [27, 29, 35, 36]. Epinephrine was given to two cases only in all patients who suffered nonanaphylactic reactions (28). Corticosteroids [35, 36, 38], antihistamines [34–36, 38], and intravenous fluids [25, 36, 38] were used in anaphylactic cases; and very few patients who suffered anaphylaxis required ventilatory support [36, 39, 40] or endotracheal intubation [39, 40]. Most of the nonanaphylactic reactions participants received corticosteroids [18, 21–23, 26, 28, 30, 31, 35] and/or antihistamines [22, 26, 28, 30–32, 35]; and few received antibiotics [22, 26, 30], H_2_ antagonists [28, 31], or antipyretics [20, 30]. No case fatalities due to anaphylactic and nonanaphylactic reactions were noted in all included studies [18–41]; and all patients had recovered or been discharged home.

### Allergen testing and SARS-CoV-2 vaccine challenge

Allergens were tested in 132 cases [21, 28, 31, 32, 34–36, 38, 41]. Skin prick tests and intradermal tests were performed in 130/132 (98.5%) and 129/132 (97.7%) cases, respectively. Only one study reported the use of Basophil Activation Test [42] which identified patient’s reactivity towards polyethylene glycol [PEG] [36]. Seventeen positive allergen skin testing were linked to the following: Pfizer-BioNTech [10, 21, 41]; polyethylene glycol [5, 38, 41]; Moderna [1, 31]; and house dust mites, peach and fruits [1, 36]. Seventy-eight negative skin testing were linked to the Pfizer-BioNTech (66) [21, 28, 34, 38, 41]; polyethylene glycol [5, 31, 32, 34, 35]; polysorbate 20 and 80 [4, 31, 34, 35, 38]; and Moderna [3, 28, 31] (Tables 1 and 2).

Two patients who had anaphylaxis following the first dose of COVID-19 vaccine successfully received the second dose without premedication or split-dosing in consultation with an allergist. These two only exhibited mild nausea and minor lip or tongue tingling on second dose administration [34, 35] (Table 1). A total of 39 study participants with reported history of nonanaphylactic reactions to COVID-19 mRNA vaccines were challenged with a second dose [21–23, 28, 35], and none were given the vaccine by split-dosing; and only 12 participants received premedication [22] and most patients then received the second dose of vaccine without a relapse of large local reactions (21–23, 28, 35). However, 3 patients had recurrent reactions similar to those after the initial dose and 3 patients had recurrent reactions that were of a lower grade than after the initial dose [22] (Table 2).

## Discussion

This study involving 26,337,421 mRNA SARS-CoV-2 vaccines recipients [Pfizer-BioNTech (n = 14,505,399) and Moderna (n = 11,831,488)] from 26 observational studies found an overall pooled prevalence estimate of anaphylaxis to both vaccines was 5.58 per million doses administered (95% CI 3.04 to 8.12), while the overall pooled prevalence estimate of nonanaphylactic reactions to both vaccines was 89.53 per million doses administered (95% CI − 11.87–190.94). Vaccination with Pfizer-BioNTech resulted in higher anaphylactic reactions compared to Moderna (9.31 per million doses administered, 95% CI 4.23–14.40 versus 3.42 per million doses administered, 95% CI 1.42–5.41), however, lower incidence of nonanaphylactic reactions was associated with Pfizer-BioNTech compared to Moderna (75.27 per million doses administered, 95% CI − 48.28–198.82 versus 99.01 per million doses administered, 95% CI − 49.77–247.80). Nevertheless, these findings on incidence rates of anaphylactic and nonanaphylactic reactions in COVID-19 mRNA vaccine recipients were limited to case reports, case series and cohort studies; and majority of studies reporting incidence rates of anaphylactic and nonanaphylactic reactions were conducted based on registry databases, and may not reflect the overall incidence rates of anaphylactic and nonanaphylactic reactions as the vast majority were from hospital and/or emergency department admission databases. We found a wide variation in the prevalences of reported anaphylactic and nonanaphylactic reactions across different studies, because they assessed different populations and settings, and used different methods. Thus, it is important that any studies in the future use a consistent and accurate definitions of anaphylactic and nonanaphylactic reactions. Use of the correct epidemiological method to define prevalence would help to identify any true difference between countries, and help to provide an overall estimate of prevalence.

Across the included studies, the most commonly identified risk factors for anaphylactic and nonanaphylactic reactions to SARS-CoV-2 mRNA vaccines were being female and previous history of atopy. Based on the published studies included in our review, evidence suggests females are much more susceptible to anaphylactic and nonanaphylactic reactions. Formation of a polyethylene glycol (PEG)-conjugated lipid derivative is hypothesized to cause COVID-19 mRNA vaccine-associated anaphylactic and nonanaphylactic reactions [44]. Sensitization to PEG is more common in women due to the relatively frequent exposure to PEG-containing products, such as cutaneous exposure to cosmetics or the use of medications such as contraceptive injections and explains female predominance in the reported cases of vaccine-associated anaphylactic and nonanaphylactic reactions [45]. Another possible explanation includes hormonal differences such as the role of estrogen which may be an important factor in allergic immunological responses [45].

Consistent with previous studies, our review found that foods, drugs or therapeutic agents, contrast media, stinging insects or jellyfish, cosmetics, detergents, household products, and latex are the most common triggers of anaphylactic and nonanaphylactic reactions induced by mRNA COVID-19 vaccines [21–23, 38, 46]. Previous history of anaphylaxis; and comorbidities such as asthma, allergic rhinitis, atopic and contact eczema/dermatitis and psoriasis and cholinergic urticaria were also described by other investigators as elicitors for anaphylactic and nonanaphylactic reactions to mRNA COVID-19 vaccines [47, 48]. Nevertheless, assessment by an allergist is NOT required for people with a history of unrelated allergies, including to allergies to foods, drugs, insect venom or environmental allergens and COVID-19 vaccines can be administered in these individuals without any special precautions [49].

The occurrence of anaphylaxis is of a particular concern in the use of newly authorized COVID-19 vaccines. The diagnosis of anaphylaxis requires professional judgment, such as case-by-case interpretation using validated diagnostic criteria for anaphylaxis [42, 43] as anaphylaxis occurrence may have a profound effect on the quality of life of the sufferer and their family [50]. It is important to identify those who might be at an increased risk of anaphylaxis in order to reduce morbidity, and provide successful management plans. Given the importance of anxiety as a contributor to the quality of life impact of COVID-19 vaccines allergy, our finding that fatal vaccine anaphylaxis incidence is relatively nil may be important information for vaccine-allergic people and their carers. Although anaphylaxis after mRNA SARS-CoV-2 vaccination is very rare, its immediate onset [usually within minutes] and life-threatening nature require that all HCWs and facilities providing vaccinations have procedures in place for anaphylaxis management. Patients who have experienced anaphylaxis should be referred to an allergy/immunology specialist to confirm the diagnosis, confirm or determine the causes(s), and determine relevant patient risk factors for severe or fatal anaphylaxis (e.g., comorbidities or concurrent medications that could be modified to reduce the patient's risk of recurrences in the future). In someone with a suspected or confirmed allergy to a SARS-CoV-2 vaccine or one of its components for whom an additional dose is required, choices include deferral of the second vaccine dose, selection of an alternative vaccine with a different platform and excipients, and the administration of the same vaccine using a graded vaccine administration protocol [51].

Healthcare workers need to differentiate the occurrence of anaphylaxis from the development of vasovagal reactions and anxiety-related symptoms [52]. While it is important to recognize and treat anaphylaxis, it is equally important not to label these other conditions as anaphylaxis, particularly when there are no objective findings. Localized cutaneous reactions were common following the mRNA vaccines and include urticarial and morbilliform eruptions which may reflect immediate hypersensitivity but have rarely been associated with anaphylaxis. There are infrequent reports of erythema multiforme, pernio/chilblains, cosmetic filler reactions, zoster, herpes simplex flares, and pityriasis rosea-like reactions, mainly occurring in high-risk patient groups [27, 30]. Ultimately, the identified cutaneous reactions are largely self-limited and should not discourage vaccination. Existing reports should reassure patients of the overall compelling safety profiles and benignity of skin reactions following the mRNA SARS-CoV-2 vaccines. Mimics of anaphylaxis should not discourage vaccination against the COVID-19 pandemic and a high rate of SARS-CoV-2 vaccines uptake across all sectors of worldwide societies is a priority public health goal. These findings should provide reassurance to HCWs and to vaccine recipients and promote confidence in the safety of COVID-19 vaccines.

### Limitations

There are several limitations to our findings. First, the quality of data submitted by reporters on anaphylactic and nonanaphylactic reactions mostly through registry databases and online questionnaires varies widely and never used standardized data collection methods. Second, most of the included studies relied on the clinical history for the diagnosis of anaphylactic and nonanaphylactic reactions. Third, some studies do not provide denominator data to calculate the incidence rates of anaphylactic and nonanaphylactic reactions; therefore, estimating accurate incidence rates by interpreting these studies databases was not possible. Fourth, the quality and completeness of the reports included in few studies might not be optimal, thus making the assessment of causality challenging. The fifth limitation is the exclusion of non-English language studies.

## Conclusion

The prevalence of COVID-19 mRNA vaccine-associated anaphylaxis is very low; and nonanaphylactic reactions occur at higher rate, however, cutaneous reactions are largely self-limited. Both anaphylactic and nonanaphylactic reactions should not discourage vaccination.

## Data Availability

Data are available upon request, please contact author for data requests.

## Abbreviations

COVID-19: Coronavirus disease 2019
IDT: Intradermal test
NOS: Newcastle–Ottawa scale
NIAID/FAAN: National Institute of Allergy and Infectious Diseases/Food Allergy and Anaphylaxis Network
PEG: Polyethylene glycol
PRISMA: Preferred Reporting Items for systematic reviews and meta-Analyses
RoB 2.0: Revised Cochrane risk of bias tool
SARS-CoV-2: Severe acute respiratory syndrome coronavirus 2
SPT: Skin prick test
VAERS: Vaccine Adverse Event Reporting System

## References

[1] World Health Organization. WHO Coronavirus (COVID-19) Dashboard 2021. https://covid19.who.int. . Accessed 28 May 2021.

[2] United States Food and Drug Administration. Pfizer-BioNTech COVID-19 Vaccine 2021. https://www.fda.gov/emergency-preparedness-and-response/coronavirus-disease-2019-covid-19/pfizer-biontech-covid-19-vaccine. Accessed 6 Jun 2021.

[3] United States Food and Drug Administration. Moderna COVID-19 Vaccine 2021. https://www.fda.gov/emergency-preparedness-and-response/coronavirus-disease-2019-covid-19/moderna-covid-19-vaccine. Accessed 6 Jun 2021.

[4] Temsah M-H, Barry M, Aljamaan F, Alhuzaimi AN, Al-Eyadhy A, Saddik B, Alsohime F, Alhaboob A, Alhasan K, Alaraj A (2021). SARS-CoV-2 B. 1.1. 7 UK variant of concern lineage-related perceptions, COVID-19 vaccine acceptance and travel worry among healthcare workers. Front Public Health.

[5] Koritala T, Hussain A, Pleshkova Y, Dondapati L, Tirupathi R, Rabaan AA, Al Mutair A, Alhumaid11 S, Al-Tawfiq12 JA, Kashyap15 R. A narrative review of emergency use authorization versus full FDA approval and its effect on COVID-19 vaccination hesitancy. 10.53854/liim-2903-4 PMC8805497 35146338

[6] Dhama K, Sharun K, Tiwari R, Dhawan M, Emran TB, Rabaan AA, Alhumaid S (2021). COVID-19 vaccine hesitancy–reasons and solutions to achieve a successful global vaccination campaign to tackle the ongoing pandemic. Hum Vaccin Immunother.

[7] American Academy of Allergy A, American College of Allergy A, Joint Council of Allergy A, Parameters JTFoP. Drug allergy: an updated practice parameter. Ann Allergy Asthma Immunol. 2010;105(4):259–73. 10.1016/j.anai.2010.08.002 20934625

[8] Lang DM, Patadia DD (2021). Anaphylaxis to vaccinations: a review of the literature and evaluation of the COVID-19 mRNA vaccinations. Cleve Clin J Med.

[9] Schnyder B, Pichler WJ (2009). Mechanisms of drug-induced allergy. Mayo Clin Proc.

[10] Su JR, Moro PL, Ng CS, Lewis PW, Said MA, Cano MV (2019). Anaphylaxis after vaccination reported to the Vaccine Adverse Event Reporting System, 1990–2016. J Allergy Clin Immunol.

[11] Moher D, Liberati A, Tetzlaff J, Altman DG, Group P (2009). Preferred reporting items for systematic reviews and meta-analyses: the PRISMA statement. PLoS Med.

[12] Peterson J, Welch V, Losos M, Tugwell P (2011). The Newcastle-Ottawa scale (NOS) for assessing the quality of nonrandomised studies in meta-analyses.

[13] Sterne JA, Savović J, Page MJ, Elbers RG, Blencowe NS, Boutron I, Cates CJ, Cheng H-Y, Corbett MS, Eldridge SM (2019). RoB 2: a revised tool for assessing risk of bias in randomised trials. BMJ.

[14] Schmidt FL, Hunter JE, Weiner IB (2003). Meta-analysis. Handbook of psychology.

[15] Higgins JP, Thompson SG (2002). Quantifying heterogeneity in a meta-analysis. Stat Med.

[16] Higgins JP, Thompson SG, Deeks JJ, Altman DG (2003). Measuring inconsistency in meta-analyses. BMJ.

[17] Egger M, Smith GD, Schneider M, Minder C (1997). Bias in meta-analysis detected by a simple, graphical test. BMJ.

[18] Ackerman M, Henry D, Finon A, Binois R, Esteve E (2021). Persistent maculopapular rash after the first dose of Pfizer-BioNTech COVID-19 vaccine. J Eur Acad Dermatol Venereol.

[19] Baden LR, El Sahly HM, Essink B, Kotloff K, Frey S, Novak R, Diemert D, Spector SA, Rouphael N, Creech CB (2021). Efficacy and safety of the mRNA-1273 SARS-CoV-2 vaccine. N Engl J Med.

[20] Bae S, Lee YW, Lim SY, Lee J-H, Lim JS, Lee S, Park S, Kim S-K, Lim Y-J, Kim EO (2021). Adverse reactions following the first dose of ChAdOx1 nCoV-19 vaccine and BNT162b2 vaccine for healthcare workers in South Korea. J Korean Med Sci.

[21] Bianchi L, Biondi F, Hansel K, Murgia N, Tramontana M, Stingeni L (2021). Skin tests in urticaria/angioedema and flushing to Pfizer-BioNTech SARS-CoV-2 vaccine: limits of intradermal testing. Allergy.

[22] Blumenthal KG, Freeman EE, Saff RR, Robinson LB, Wolfson AR, Foreman RK, Hashimoto D, Banerji A, Li L, Anvari S (2021). Delayed large local reactions to mRNA-1273 vaccine against SARS-CoV-2. N Engl J Med.

[23] Corbeddu M, Diociaiuti A, Vinci M, Santoro A, Camisa V, Zaffina S, El Hachem M (2021). Transient cutaneous manifestations after administration of Pfizer-BioNTech COVID-19 Vaccine: an Italian single centre case series. J Eur Acad Dermatol Venereol.

[24] Covid C, Team R (2021). Allergic reactions including anaphylaxis after receipt of the first dose of Pfizer-BioNTech COVID-19 vaccine—United States, December 14–23, 2020. Morb Mortal Wkly Rep.

[25] Frank A, Radparvar S, Manasia A, Bassily-Marcus A, Kohli-Seth R (2021). Prolonged anaphylaxis to pfizer coronavirus disease 2019 vaccine: a case report and mechanism of action. Crit Care Explor.

[26] Johnston MS, Galan A, Watsky KL, Little AJ (2021). Delayed localized hypersensitivity reactions to the moderna COVID-19 vaccine: a case series. JAMA Dermatol.

[27] Kadali RAK, Janagama R, Peruru S, Gajula V, Madathala RR, Chennaiahgari N, Malayala SV (2021). Adverse effects of COVID-19 mRNA-1273 vaccine: a randomized, cross-sectional study on healthcare workers with detailed self-reported symptoms. J Med Virol.

[28] Kelso JM (2021). Misdiagnosis of systemic allergic reactions to mRNA COVID-19 vaccines. Ann Allergy Asthma Immunol.

[29] Mathioudakis AG, Ghrew M, Ustianowski A, Ahmad S, Borrow R, Papavasileiou LP, Petrakis D, Bakerly ND (2021). Self-reported real-world safety and reactogenicity of COVID-19 vaccines: a vaccine recipient survey. Life.

[30] McMahon DE, Amerson E, Rosenbach M, Lipoff JB, Moustafa D, Tyagi A, Desai SR, French LE, Lim HW, Thiers BH (2021). Cutaneous reactions reported after moderna and pfizer COVID-19 vaccination: a registry-based study of 414 cases. J Am Acad Dermatol.

[31] Mustafa SS, Ramsey A, Staicu ML (2021). Administration of a second dose of the moderna COVID-19 vaccine after an immediate hypersensitivity reaction with the first dose: two case reports. Ann Intern Med.

[32] Ocáriz M, Zubeldia Ortuño J (2021). Safety of new MRNA vaccines against COVID-19 in severe allergic patients. J Investig Allergol Clin Immunol.

[33] Ontario Agency for Health Protection and Promotion (Public Health Ontario). Reports of events managed as anaphylaxis following COVID-19 vaccines in Ontario: December 13, 2020 to March 6, 2021 2021. https://www.publichealthontario.ca/-/media/documents/ncov/epi/covid-19-anaphylaxis-epi-summary.pdf?la=en. Accessed 29 May 2021.

[34] Park LHJ, Montgomery CJR, Boggs LNA (2021). Anaphylaxis after the Covid-19 vaccine in a patient with cholinergic urticaria. Mil Med.

[35] Pitlick MM, Sitek AN, Kinate SA, Joshi AY, Park MA (2021). Polyethylene glycol and polysorbate skin testing in the evaluation of COVID-19 vaccine reactions: early report. Ann Allergy Asthma Immunol.

[36] Restivo V, Candore G, Barrale M, Caravello E, Graziano G, Onida R, Raineri M, Tiralongo S, Brusca I (2021). Allergy to polyethilenglicole of anti-SARS CoV2 vaccine recipient: a case report of young adult recipient and the management of future exposure to SARS-CoV2. Vaccines.

[37] Riad A, Pokorná A, Attia S, Klugarová J, Koščík M, Klugar M (2021). Prevalence of COVID-19 Vaccine Side Effects among Healthcare Workers in the Czech Republic. J Clin Med.

[38] Sellaturay P, Nasser S, Islam S, Gurugama P, Ewan PW (2021). Polyethylene glycol (PEG) is a cause of anaphylaxis to the Pfizer/BioNTech mRNA COVID-19 vaccine. Clin Exp Allergy.

[39] Shimabukuro T (2021). Allergic reactions including anaphylaxis after receipt of the first dose of Moderna COVID-19 vaccine—United States, December 21, 2020–January 10, 2021. Am J Transplant.

[40] Shimabukuro T, Nair N (2021). Allergic reactions including anaphylaxis after receipt of the first dose of Pfizer-BioNTech COVID-19 vaccine. JAMA.

[41] Vieira J, Marcelino J, Ferreira F, Farinha S, Silva R, Proença M, Tomaz E (2021). Skin testing with Pfizer SARS-CoV-2 vaccine and PEG 2000. Asia Pac Allergy.

[42] Rüggeberg JU, Gold MS, Bayas J-M, Blum MD, Bonhoeffer J, Friedlander S, de Souza BG, Heininger U, Imoukhuede B, Khamesipour A (2007). Anaphylaxis: case definition and guidelines for data collection, analysis, and presentation of immunization safety data. Vaccine.

[43] Sampson HA, Muñoz-Furlong A, Campbell RL, Adkinson NF, Bock SA, Branum A, Brown SG, Camargo CA, Cydulka R, Galli SJ (2006). Second symposium on the definition and management of anaphylaxis: summary report—Second National Institute of Allergy and Infectious Disease/Food Allergy and Anaphylaxis Network symposium. Journal of Allergy and Clinical Immunology.

[44] Castells MC, Phillips EJ (2021). Maintaining safety with SARS-CoV-2 vaccines. N Engl J Med.

[45] Somiya M, Mine S, Yasukawa K, Ikeda S (2021). Sex differences in the incidence of anaphylaxis to LNP-mRNA COVID-19 vaccines. Vaccine.

[46] Rutkowski K, Mirakian R, Till S, Rutkowski R, Wagner A (2021). Adverse reactions to COVID-19 vaccines: a practical approach. Clin Exp Allergy.

[47] Dages KN, Pitlick MM, Joshi AY, Park MA (2021). Risk of allergic reaction in patients with atopic disease and recent COVID-19 vaccination. Ann Allergy Asthma Immunol.

[48] Krantz MS, Bruusgaard-Mouritsen MA, Koo G, Phillips EJ, Stone CA, Garvey LH (2021). Anaphylaxis to the first dose of mRNA SARS-CoV-2 vaccines: don’t give up on the second dose. Allergy.

[49] Vander Leek TK, Chan ES, Connors L, Derfalvi B, Ellis AK, Upton JE, Abrams EM (2021). COVID-19 vaccine testing & administration guidance for allergists/immunologists from the Canadian Society of Allergy and Clinical Immunology (CSACI). Allergy Asthma Clin Immunol.

[50] Panesar SS, Nwaru BI, Hickstein L, Rader T, Hamadah H, Ali DFI, Patel B, Muraro A, Roberts G, Worm M (2013). The epidemiology of anaphylaxis in Europe: protocol for a systematic review. Clin Transl Allergy.

[51] Canadian Society of Allergy and Clinical Immunology (CSACI). SARS-CoV-2 vaccine testing & administration guidance for allergists/immunologists from the CSACI 2021. https://csaci.ca/wp-content/uploads/2021/04/2021-04-10-UPDATE-COVID-19-Vaccine-Testing-Administration-Guidance.pdf. Accessed 26 Sep 2021.

[52] Gold MS, MacDonald NE, McMurtry CM, Balakrishnan MR, Heininger U, Menning L, Benes O, Pless R, Zuber PL (2020). Immunization stress-related response–redefining immunization anxiety-related reaction as an adverse event following immunization. Vaccine.

[53] Altrichter S, Salow J, Ardelean E, Church MK, Werner A, Maurer M (2014). Development of a standardized pulse-controlled ergometry test for diagnosing and investigating cholinergic urticaria. J Dermatol Sci.

